# Four Routes to 3-(3-Methoxy-1,3-dioxopropyl)pyrrole, a Core Motif of Rings C and E in Photosynthetic Tetrapyrroles

**DOI:** 10.3390/molecules28031323

**Published:** 2023-01-30

**Authors:** Khiem Chau Nguyen, Anh Thu Nguyen Tran, Pengzhi Wang, Shaofei Zhang, Zhiyuan Wu, Masahiko Taniguchi, Jonathan S. Lindsey

**Affiliations:** Department of Chemistry, North Carolina State University, Raleigh, NC 27695, USA

**Keywords:** acylation, bacteriochlorophyll, β-ketoester, Boc-pyrrole, carbonylation, chlorophyll, synthesis, TIPS-pyrrole

## Abstract

The photosynthetic tetrapyrroles share a common structural feature comprised of a β-ketoester motif embedded in an exocyclic ring (ring E). As part of a total synthesis program aimed at preparing native structures and analogues, 3-(3-methoxy-1,3-dioxopropyl)pyrrole was sought. The pyrrole is a precursor to analogues of ring C and the external framework of ring E. Four routes were developed. Routes 1–3 entail a Pd-mediated coupling process of a 3-iodopyrrole with potassium methyl malonate, whereas route 4 relies on electrophilic substitution of TIPS-pyrrole with methyl malonyl chloride. Together, the four routes afford considerable latitude. A long-term objective is to gain the capacity to create chlorophylls and bacteriochlorophylls and analogues thereof by facile de novo means for diverse studies across the photosynthetic sciences.

## 1. Introduction

**Chart 1:**
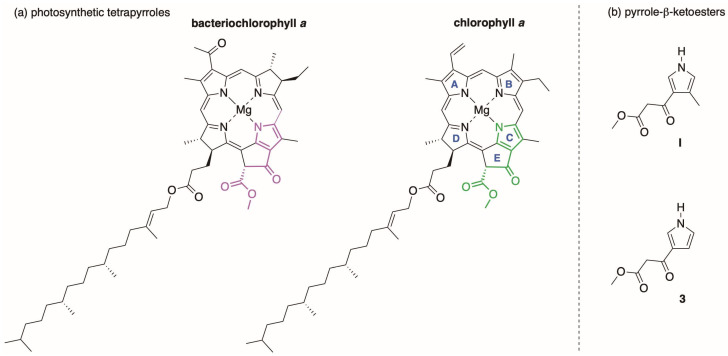
Representative photosynthetic tetrapyrroles (**a**) and pyrrole-β-ketoesters (**b**).

Photosynthetic tetrapyrroles harvest sunlight and thereby channel the energy that drives the biosphere, yet the chemical synthesis of such molecules—an enabling methodology for diverse studies—has largely been neglected [1]. While the structures within the family of bacteriochlorophylls and chlorophylls vary in terms of the extent of saturation and nature of peripheral substituents [2], selected structural motifs are constant across the family. One example in this regard is the fifth ring, known equivalently as ring E, the exocyclic ring, or the isocyclic ring. The structures of bacteriochlorophyll *a* and chlorophyll *a*, the chief pigments of anoxygenic and oxygenic photosynthesis, respectively, are shown in Chart 1.

**Scheme 1:**
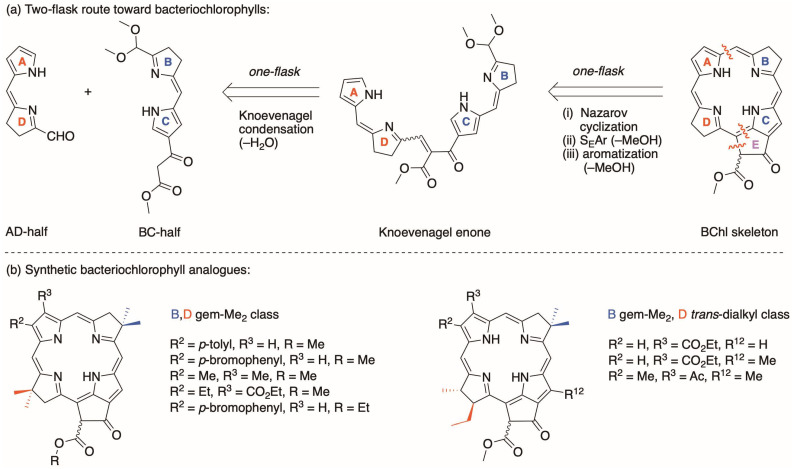
Retrosynthesis for bacteriochlorophylls and analogues (**a**), with omission of substituents for clarity. Macrocycles prepared in this manner (**b**).

We are working toward synthetic strategies that provide access to the entire suite of native photosynthetic tetrapyrroles as well as synthetic analogues [3,4,5,6,7,8]. The general route under development is shown in Scheme 1 panel a. An AD-half and a BC-half undergo Knoevenagel condensation [9] to form the corresponding Knoevenagel enone. The enone then undergoes a double-ring closure process via Nazarov cyclization [10] (forming ring E), electrophilic aromatic substitution (S_E_Ar, forming the macrocycle), and elimination of methanol, giving the aromatic macrocycle. The conditions for the Knoevenagel condensation entail 40 mM reactants (AD-half, BC-half) in acetonitrile containing piperidine, acetic acid, and molecular sieves at room temperature (overnight). The recently optimized conditions [7] for the one-flask, double-ring closure entail 0.20 mM reactant (Knoevenagel enone) in acetonitrile containing Yb(OTf)_3_ at 80 °C (4 h). The Knoevenagel condensation and the double-ring closure process each affords a yield of ≈50%. To date, eight bacteriochlorophyll analogues have been prepared in this general manner, as shown in Scheme 1 panel b [3,5,6,7]. Three chlorophyll analogues also have been prepared by employing a dipyrromethane BC-half (not shown) [4].

In early synthetic routes toward native photosynthetic tetrapyrroles and analogues thereof, formation of ring E presented a challenge that was met by annulation of an intact macrocycle [11]. In the route shown in Scheme 1, ring E is formed in a process integral to macrocycle formation. The nascent ring E is the nexus for macrocycle formation, and the β-ketoester attached to ring C is the linchpin. The β-ketoester motif at the 3-position participates in both Knoevenagel and Nazarov reactions to form ring E in a double-ring closure process [3,5,6,7,8]. Hence, access to diverse β-ketoester-bearing ring C precursors is an important sub-objective of the overall synthesis program.

We recently described the synthesis of 3-(3-methoxy-1,3-dioxopropyl)-4-methylpyrrole (**I**) [4], a precursor to native ring C that bears the requisite β-ketoester motif (Chart 1). For fundamental studies of the core chromophores undecorated with peripheral substituents [11], we sought the analogue of **I** that lacks the 4-methyl substituent (**3**). A single methyl group can increase the reactivity of a pyrrole toward electrophiles by ≈30-fold [12,13]. On the other hand, sparsely substituted tetrapyrroles often exhibit spectral features altered from those of the native macrocycles (vide infra), and the open β-pyrrole positions provide sites for unwanted reactivity [14].

Inspection of the macrocycles that we have prepared to date shows some control over the presence of the substituent at the 12-position (Scheme 1, panel b) and might suggest the issue of the 12-H or 12-Me group is a settled matter. The development of the route initially employed a gem-dimethyl group in each pyrroline ring in lieu of the native-*trans*-dialkyl group (Scheme 1, panel b). Each **BC-half** bearing a gem-dimethyl group was synthesized by installation of the β-ketoester motif in a fairly late-stage, Pd-mediated coupling process [3,4,8]. In contrast, the presently envisaged synthesis of a **BC-half** bearing a native-*trans*-dialkyl group requires early installation of the β-ketoester motif.

In this paper, we report four routes to **3**. Routes 1–3 entail a Pd-mediated coupling process of a 3-iodopyrrole with potassium methyl malonate, whereas route 4 relies on electrophilic substitution of TIPS-pyrrole with methyl malonyl chloride. The various routes afford considerable latitude depending on available starting materials, desired yield, and other constraints. As part of this Special Issue on Macrocycles, we also outline some of the motivations for the preparation of tailored bacteriochlorophylls and chlorophylls, particularly sparsely substituted macrocycles that would make use of pyrrole **3**.

## 2. Reconnaissance

**Scheme 2:**
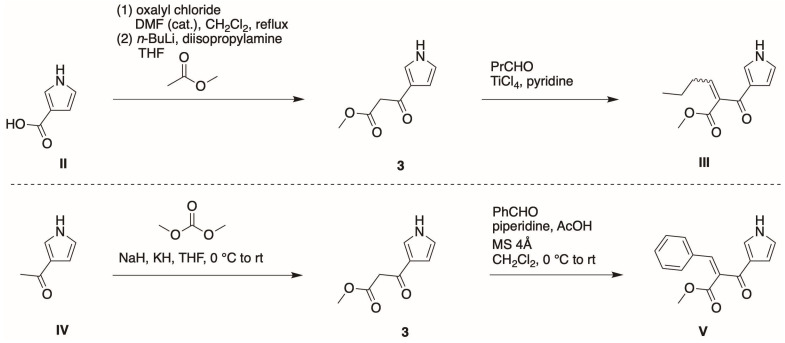
Prior reports concerning **3**.

Pyrrole **3** has been employed in studies of Knoevenagel condensation and subsequent Nazarov reaction, but without experimental procedures, characterization data, or information about scale. Two routes have been alluded to for the preparation of **3** (Scheme 2) [15,16].

Malona et al., referring to general procedures of others [17,18], indicated that pyrrole-3-carboxylic acid (**II**) was converted to the acid chloride, which upon reaction with the enolate of methyl acetate affords **3** (Scheme 2) [15]. Subsequent Knoevenagel condensation [19] with butanal gave **III**. Fujiwara et al. reported a specific procedure for 2-acetylpyrrole, which was then employed for reaction of 3-acetylpyrrole (**IV**) [16]. Thus, the enolate of 3-acetylpyrrole (**IV**) was reacted with dimethyl carbonate to obtain **3** [16], which upon subsequent Knoevenagel condensation with benzaldehyde gave **V** [16]. The overall yield in the two-step process was 35%. In neither case were specific experimental procedures, yield values, or characterization data provided for **3**. The chief focus of the two research groups was to probe the Nazarov reactions with a variety of heterocycles [15] or to examine diverse catalysts [16]; in each case, extensive studies were reported with 2-substituted pyrroles, whereas **3** was used in only a single instance. The 3-substituted pyrroles **II** and **IV** are ≈30 and ≈300 times more expensive than the 2-substituted pyrroles, respectively, which may have influenced the choice of substrates for investigation.

## 3. Results

### 3.1. Synthetic Routes to a Pyrrole-β-Ketoester

**Scheme 3:**
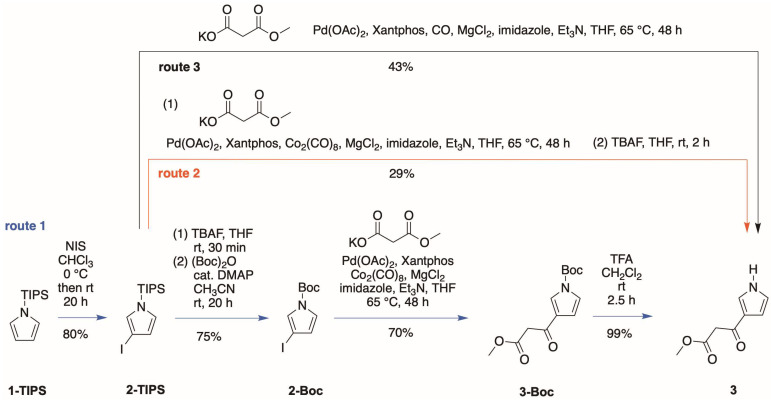
Three routes to the target pyrrole **3**.

Route 1 begins with commercially available 1-(triisopropylsilyl)pyrrole (**1-TIPS**) [20], which upon treatment with *N*-iodosuccinimide (NIS) in anhydrous CHCl_3_ afforded β-iodopyrrole **2-TIPS** in 80% yield (Scheme 3, blue arrow). The compound **2-TIPS** has been prepared previously from **1-TIPS** at large scale by bromo-lithium exchange [20], use of I_2_ [21], and use of NIS in acetone [22]. Our prior studies [4] showed that a Boc-protected halopyrrole is superior to a TIPS-protected halopyrrole in carbonylation to install the β-ketoester group. Thus, treatment of **2-TIPS** with tetra-*n*-butylammonium fluoride (TBAF) followed by *tert*-butoxycarbonyl anhydride [(Boc)_2_O] and a catalytic amount of 4-dimethylaminopyridine (DMAP) gave the known [23,24] Boc-protected analogue **2-Boc** in 75% yield. Note that the unmasked 3-iodopyrrole was quite unstable (toward light, air, moisture, and acid) and began to decompose shortly after purification, consistent with the general sensitivity of halopyrroles [25]. The TIPS/Boc protecting group not only afforded excellent selectivity of halogenation and carbonylation but also increased the stability and scalability for manipulation of this type of compound.

The installation of the β-ketoester relies on a known reaction of an aryl halide and potassium monomethyl malonate [26] that we have used previously with other pyrroles [3,4,5,8]. Here, the reaction of 3-iodopyrrole **2-Boc** and potassium monomethyl malonate was carried out in tetrahydrofuran (THF) containing Pd(OAc)_2_, Xantphos, Co_2_(CO)_8_, MgCl_2_, imidazole, and triethylamine at 65 °C for 48 h. In this manner, the corresponding pyrrole-β-ketoester **3-Boc** was obtained in 70% yield. Finally, dissolution of **3-Boc** in CH_2_Cl_2_ containing trifluoroacetic acid (TFA) afforded the desired **3** in 99% yield (Scheme 3, blue arrow).

Route 2 employs the palladium-mediated carbonylation of **2-TIPS** to install the β-ketoester motif. Following workup, cleavage of the TIPS group by treatment with TBAF afforded the desired compound **3** in 29% yield (Scheme 3, red arrow).

Route 3 affords a simplification of route 2. Routes 1 and 2 both employed Co_2_(CO)_8_ as a source of carbon monoxide. Carbon monoxide is essential as the carbon constitutes the carbonyl that is attached to the pyrrole 3-position [26]. Upon use of CO gas instead of Co_2_(CO)_8_ under the same conditions for carbonylative coupling of **2-TIPS** with potassium monomethyl malonate [26], we were surprised to find that the target compound **3** formed directly in 43% yield. We have no explanation for this observation, but the higher yield, avoidance of the costly reagents Co_2_(CO)_8_ and TBAF, and lack of a second workup procedure collectively comprise a serendipitous finding.

**Scheme 4:**
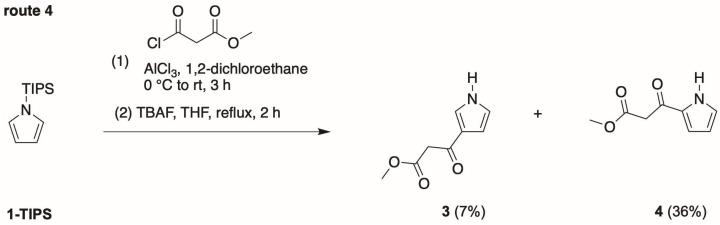
Streamlined synthesis (route 4) of the target pyrrole **3**.

Route 4 arose from a desire for even greater simplicity. Friedel–Crafts acylation [27] of pyrrole **1-TIPS** was carried out with methyl malonyl chloride in 1,2-dichloroethane containing AlCl_3_ (Scheme 4). The conditions are very similar to those of Bray et al. [21], who reported the Friedel–Crafts acylation of pyrrole **1-TIPS** with a variety of acylating agents. Subsequent treatment to TBAF in refluxing THF gave the desired pyrrole **3** along with the 2-substituted isomer **4**, a known [15,16,28] compound. In one preparation (12 mmol of **1-TIPS**), the respective yields were 15% and 14%. In a second preparation (100 mmol of **1-TIPS**), the respective yields were 7% (1.10 g) and 36% (6.16 g). The two isomers were readily separable by column chromatography, evidenced by the clean separation on thin layer chromatography (TLC) (R_f_ = 0.21 and 0.42 with hexanes/ethyl acetate (1:1) on silica). The latter preparation was repeated once, with identical results.

The formation of the 2-substituted isomer (**4**) was surprising because the TIPS group is designed to block reaction by steric hindrance at the 2-position, causing reaction to proceed preferentially at the 3-position. Several experiments were carried out to explore this result.

**Scheme 5:**
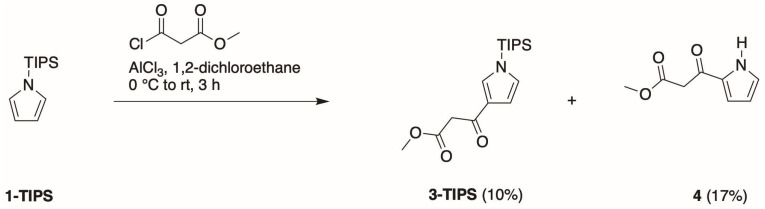
Friedel–Craft acylation of **1-TIPS**.

- First, treatment of pyrrole with methyl malonyl chloride in the presence of AlCl_3_ in 1,2-dichloroethane at 0 °C to room temperature for 3 h gave the 2-substituted isomer (**4**) in 30% isolated yield.
- Second, Bray and coworkers [21] reported that TIPS-pyrrole would react with potent acyl electrophiles in the absence of a Lewis acid. In our hands, the reaction of **1-TIPS** with methyl malonyl chloride in 1,2-dichloroethane containing pyridine at 0 °C to reflux for 24 h gave two unknown products.
- Third, the same reaction of **1-TIPS** with methyl malonyl chloride in nitromethane containing one of several Lewis acids also afforded **4** as the only product detectable by ^1^H-NMR spectroscopy. The Lewis acids were those identified in reactions of heterocycles [29], namely, Ga(OTf)_3_, Yb(OTf)_3_, and Hf(OTf)_3_. The results indicated that the TIPS group had been removed during the course of the reaction, without any treatment with a fluoride reagent, stemming most likely from chloride liberated during the course of the reaction.
- Fourth, we again treated **1-TIPS** with methyl malonyl chloride in 1,2-dichloroethane containing AlCl_3_ (Scheme 5). The two products isolated were **3-TIPS** and **4**; thus, 3-acylation occurred with the TIPS group intact, whereas it is most likely that 2-acylation occurred following the adventitious loss of the TIPS group. The subsequent treatment with TBAF, as shown in Scheme 4, then is only required for the conversion of **3-TIPS** to the pyrrole **3**. To achieve a higher yield in the acylation of **3-TIPS** will require identification of reaction conditions wherein the TIPS group remains intact, thereby thwarting 2-acylation. Regardless, route 4 as is remains the most expeditious among the four routes examined. The synthesis of **3-TIPS** was achieved by independent means, as described in the next section.

### 3.2. Structural Studies

**Figure 1:**
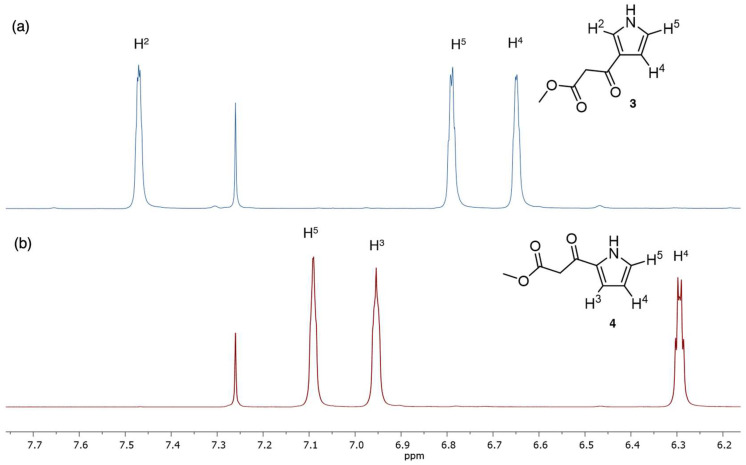
^1^H-NMR spectral region of two isomers (in CDCl_3_ at room temperature): (**a**) the 3-substituted pyrrole (**3**) and (**b**) the 2-substituted pyrrole (**4**).

The identities of **3** and **4** are readily distinguished by ^1^H-NMR spectroscopy. The ^1^H-NMR spectra in CDCl_3_ are shown in Figure 1. 

Each pyrrole contains three C–H bonds and shows three multiplets (Figure 1). The wide chemical shifts of three protons provide a clear distinction and basis for structural assignment. The assignments for **4** were based on COSY measurements (H^4^) and NOESY measurements (H^3^ interaction with the ketoester methylene moiety). The assignments for **3** were based exclusively on NOESY measurements for H^2^ (interaction with the ketoester methylene moiety and with the N–H proton) and H^5^ (interaction with the N–H proton), with H^4^ assigned by difference. The chemical shift of the N–H proton, while less reliable, was at 8.7, 9.0, or 9.1 ppm for **3** (three samples) versus ≈9.8 ppm for **4**. The clear distinctions in spectra make disambiguation of the two isomers clear.

**Figure 2:**
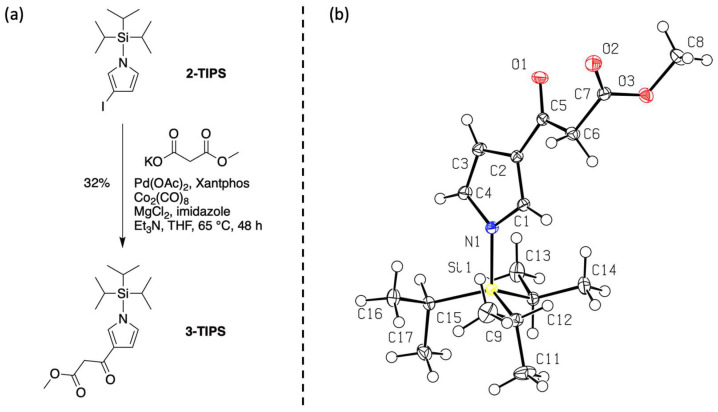
Installation of the β-ketoester (**a**) and ORTEP diagram of **3-TIPS** with thermal ellipsoids drawn at the 50% probability level (**b**).

The target compound **3** is an oil at room temperature. The Pd-mediated carbonylation of **2-TIPS** was carried out using Co_2_(CO)_8_ but without subsequent treatment with TBAF (Figure 2). The TIPS derivative **3-TIPS** was isolated as a solid at room temperature (m.p. 76–78 °C). A single-crystal X-ray crystallographic structure was obtained, verifying formation of the β-ketoester scaffold (Figure 2). The size of the TIPS group is comparable to that of the pyrrole-β-ketoester. The structure clearly shows the hindrance by the TIPS group imparted to the 2,5-positions versus the 3,4-positions of the pyrrole molecule. Additional X-ray crystallographic data are provided in the Supplementary Materials.

## 4. Discussion

In this section, we first compare the four routes explored herein for the synthesis of **3**. We then turn to consider part of the rationale for the *de novo* synthesis of analogues of native bacteriochlorophylls and chlorophylls.

### 4.1. Comparison of Routes

A long-term objective is to establish synthetic methodology that enables access to native photosynthetic macrocycles and analogues. Such syntheses present a significant number of challenges beyond obvious issues of achieving the desired patterns of substituents, stereochemistry, and pyrrole versus pyrroline rings. One challenge is scale: we seek ≥ 5 mmol of each precursor to rings A–D, 0.5 mmol of each **AD-half** and **BC-half**, and 0.05 mmol of each target macrocycle. A second challenge is efficacy: we seek procedures such that a skilled investigator, with individual A–D ring precursors in hand, can create one macrocycle in one month.

The four routes described here afford considerable latitude in choice of synthetic approach to a precursor to an analogue of ring C. Conversion of pyrrole **1-TIPS** to **3** could be achieved in four steps in 42% yield (route 1), in three steps in 23% yield (route 2), and in two steps via the intermediacy of **2-TIPS** in 34% yield (route 3). Routes 1–3 rely on the intermediacy of **2-TIPS**. The conversion of pyrrole **1-TIPS** to **3** was obtained in two steps in 7 or 15% yield (route 4) without the intermediacy of **2-TIPS** and the use of a Pd-mediated coupling reaction. Route 4 places expediency over elegance yet readily afforded 1.1 g (6.6 mmol) of **3**. The use of Friedel–Crafts acylation in route 4 harkens back to an era of organic chemistry that long predates the use of Pd-mediated coupling reactions, but is attractive in its simplicity and efficacy. Facile access to >5 mmol of **3** satisfies the objective for extension to the synthesis of native photosynthetic tetrapyrroles and analogues.

### 4.2. Perspective on Design

The proper functioning of bacteriochlorophylls and chlorophylls in native photosynthetic machinery requires appropriate energetic features of each macrocycle. The energetics comes in two flavors: (1) An appropriate position of the long-wavelength absorption band of each macrocycle, which in large part determines the excited-state energy level. Control of the latter parameter is essential for flow of excited-state energy in the antennas and from the antenna to the reaction centers. (2) An appropriate level of the highest-occupied molecular orbital (HOMO) and lowest-unoccupied molecular orbital (LUMO) for those molecules to facilitate, or preclude, electron-transfer processes. The electron transfer from one photosynthetic pigment to another generates a hole in the HOMO of the donor and single occupancy of the former LUMO of the acceptor. Control over redox potentials is essential for efficient charge-separation processes.

**Figure 3:**
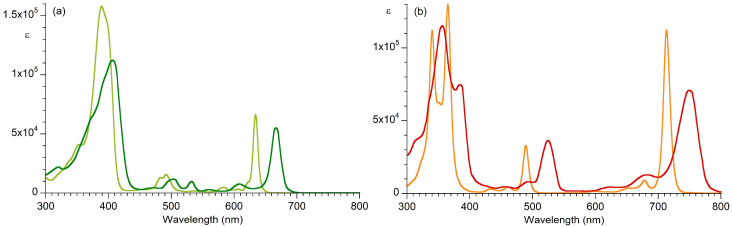
Absorption spectra at room temperature. (**a**) **H_2_C** (in toluene, lime) [30,33] and **pheophytin *a*** (in Et_2_O, green) [32]. (**b**) **H_2_BC** (in toluene, orange) [31] and **bacteriopheophytin *a*** (in benzene, red) [32].

**Chart 2:**
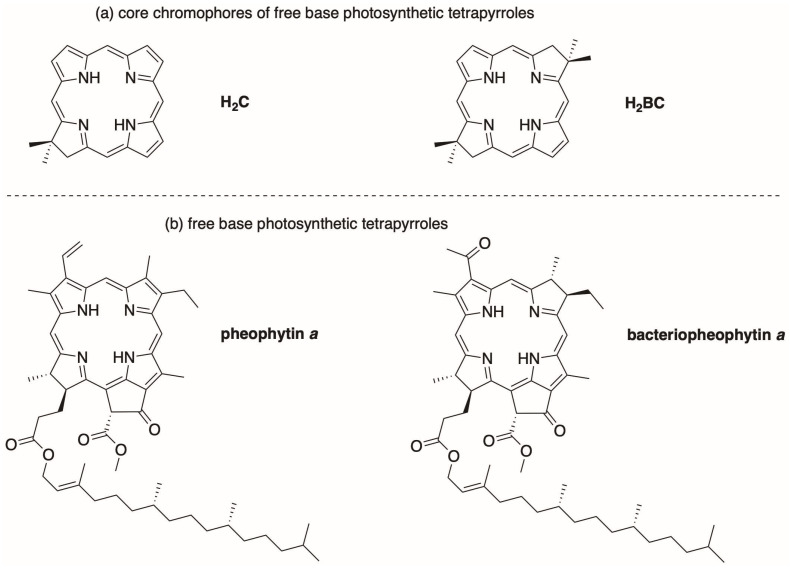
Free base macrocycles. Synthetic chlorin **H_2_C** and bacteriochlorin **H_2_BC** (**a**) and native macrocycles (**b**).

The absorption spectra of two native photosynthetic tetrapyrrole macrocycles, **bacteriopheophytin *a*** and **pheophytin *a*** (where “pheo” implies the free base macrocycle), are shown in Figure 3. The spectra are compared with those of core macrocycles that lack any substituents other than a gem-dimethyl group in each pyrroline ring, namely, the chlorin **H_2_C** [30] and the bacteriochlorin **H_2_BC** [31]. The structures of the four macrocycles are shown in Chart 2. The core macrocycles lack ring E. The parameters corresponding to the spectra are listed in Table 1. The parameters for each band include the position of wavelength maximum (λ_abs_, in nm), the full-width-at-half-maximum (fwhm, in nm), and molar absorption coefficient (ε, in M^−1^cm^−1^). The spectra were obtained from the literature [30,31,32,33]. The spectra of additional native and synthetic macrocycles are available in PhotochemCAD [33,34,35].

The substituents arrayed about the perimeter of the native macrocycles serve as auxochromes [36]. The spectra of the native macrocycles can be compared with those of the naked synthetic counterparts. Painting with a broad brush, the net effect of the substituents for each macrocycle causes oscillator strength to be shifted from the near-ultraviolet (B) band to the long-wavelength Q_y_ band. The effects of the potent auxochromes such as vinyl, acetyl, and the keto group of ring E are reasonably well known (as such groups are readily modifiable) [37], but much less is known about the effects of individual methyl groups. Moreover, while prediction of spectral properties is a matter under active investigation by calculation [38,39,40], much less is known about electrochemical properties [41]. Both spectra, which reflect the difference in energy of ground and excited-states, and energies of anion radicals and cation radicals in solution (for which calculated HOMO and LUMO energies provide only an estimate), bear critically on photosynthetic function. The ability to synthesize analogues of the native macrocycles would enable a variety of fundamental studies in this regard.

## 5. Materials and Methods

### 5.1. General Methods

^1^H-NMR and ^13^C{^1^H}-NMR spectra were collected at room temperature in CDCl_3_ unless noted otherwise. The recorded NMR spectra of the compounds can be found in the Supplementary Materials. Electrospray ionization mass spectrometry (ESI-MS) data are reported for the molecular ion or protonated molecular ion. Silica gel (40 μm average particle size) was used for column chromatography. THF used in all reactions was freshly distilled from Na/benzophenone ketyl unless noted. All commercially available compounds were used as received. The Co_2_(CO)_8_ was received as a solid stabilized with hexane, whereupon the content of the cobalt complex was 95% by mass. The number of mmol employed in each reaction has been corrected accordingly from the weighed quantity.

### 5.2. Synthesis and Characterization

#### 5.2.1. Route 1

*3-Iodo-1-(triisopropylsilyl)pyrrole* (**2-TIPS**) [20,21,22]. Following a standard procedure [4], a solution of **1-TIPS** (2.23 g, 10.0 mmol) in CHCl_3_ (20 mL, anhydrous with stabilization by amylenes) at 0 °C under argon was treated with NIS (2.24 g, 10.0 mmol). The reaction flask was covered with aluminum foil. The reaction mixture was stirred for 20 h at room temperature before washing with 20% aqueous Na_2_S_2_O_3_ solution. The organic layer was separated, dried (Na_2_SO_4_), and concentrated to a yellow oil, which was passed through a neutral aluminum oxide pad eluting with hexanes to afford a colorless liquid (2.78 g, 80%): ^1^H-NMR (600 MHz, CDCl_3_) δ 1.09 (d, *J* = 7.5 Hz, 18H), 1.39–1.44 (m, 3H), 6.36 (dd, *J* = 2.7 and 1.3 Hz, 1H), 6.66 (t, *J* = 2.5 Hz, 1H), 6.79 (dd, *J* = 2.2 and 1.4 Hz, 1H); ^13^C{^1^H}-NMR (150 MHz, CDCl_3_) δ 11.6, 17.7, 62.2, 117.5, 125.7, 128.7; HRMS (ESI-TOF) *m/z*: [M + H]^+^ calcd. for C_13_H_25_INSi 350.0796; found 350.0802.

*1-(tert-Butoxycarbonyl)-3-iodopyrrole* (**2-Boc**) [23,24]. Following a standard procedure [4], a solution of **2-TIPS** (699 mg, 2.00 mmol) in THF (2.0 mL, ACS grade) was treated with TBAF (3.0 mL, 1 M in THF, 3 mmol) at room temperature. The reaction progress was monitored by TLC, and after 30 min, the reaction was completed. The reaction mixture was washed with water and brine, extracted with ethyl acetate, dried (Na_2_SO_4_), and concentrated. The resulting yellow oil (silica TLC, R_f_ = 0.61 with hexanes/ethyl acetate (3:1)) was passed through a silica pad (hexanes/ethyl acetate (20:1)) to afford a colorless oil. The resulting oil was dissolved in acetonitrile (20 mL, ACS grade) and then was treated with (Boc)_2_O (1.31 g, 6.0 mmol) and DMAP (25 mg, 0.20 mmol) at room temperature. The resulting reaction mixture was stirred for 20 h before washing with brine. The organic layer was separated, dried (Na_2_SO_4_), and concentrated. The resulting orange oil (silica TLC, R_f_ = 0.28 with hexanes/CH_2_Cl_2_ (10:1)) was purified by column chromatography (silica, hexanes/ethyl acetate (20:1)) to afford a colorless oil (441 mg, 75%): ^1^H-NMR (600 MHz, CDCl_3_) δ 1.59 (s, 9H), 6.28 (dt, *J* = 3.2 and 1.4 Hz, 1H), 7.11–7.14 (m, 1H), 7.31 (d, *J* = 1.8 Hz, 1H); ^13^C{^1^H}-NMR (150 MHz, CDCl_3_) δ 27.9. 65.2, 84.4, 118.8, 121.4, 124.7, 147.5; HRMS (ESI-TOF) *m/z*: [M + H]^+^ calcd. for C_9_H_13_INO_2_ 293.9986; found 293.9983.

*1-tert-Butoxycarbonyl-3-(3-methoxy-1,3-dioxopropyl)pyrrole* (**3-Boc**). Following a reported procedure [3], **2-Boc** (131 mg, 0.450 mmol), methyl potassium malonate (112 mg, 0.720 mmol), Xantphos (156 mg, 0.270 mmol), MgCl_2_ (69 mg, 0.72 mmol), and imidazole (61 mg, 0.90 mmol) were placed in a Schlenk flask, which was charged with argon. THF (9.0 mL) was added followed by triethylamine (97 µL, 0.70 mmol). The mixture was degassed by three freeze–pump–thaw cycles. Then, Pd(OAc)_2_ (117 mg, 0.52 mmol) and Co_2_(CO)_8_ (178 mg, 0.49 mmol) were added. The flask was sealed immediately and heated at 65 °C in an oil bath for two days, and then the reaction mixture was allowed to cool to room temperature. The mixture was diluted with ethyl acetate and then filtered through a Celite pad. The filtrate was washed with brine and water, dried (Na_2_SO_4_), and concentrated. The crude mixture was purified by column chromatography (silica, hexanes/ethyl acetate (6:1)) wherein the second fraction was isolated (silica TLC, R_f_ = 0.53 with hexanes/ethyl acetate (3:1)) as a yellow oil (83 mg, 70%): ^1^H-NMR (600 MHz, CDCl_3_) δ 1.62 (s, 9H), 3.75 (s, 3H), 3.80 (s, 2H), 6.64 (dd, *J* = 3.4 Hz and 1.7 Hz, 1H), 7.23 (dd, *J* = 3.4 and 2.0 Hz, 1H), 7.85 (t, *J* = 1.9 Hz, 1H); ^13^C{^1^H}-NMR (150 MHz, CDCl_3_) δ 27.9, 46.6, 52.5, 85.6, 111.0, 121.6, 125.1, 127.3, 148.0, 167.8, 187.2; HRMS (ESI-TOF) *m/z*: [M + H]^+^ calcd. for C_13_H_18_NO_5_ 268.1180; found 268.1173.

*3-(3-Methoxy-1,3-dioxopropyl)pyrrole* (**3**). Following a standard procedure [5], a solution of **3-Boc** (58 mg, 0.22 mmol) in CH_2_Cl_2_ (2.0 mL, ACS grade) was treated dropwise with a solution of TFA (250 µL) in CH_2_Cl_2_ (250 µL, ACS grade) at room temperature. The reaction mixture was stirred at room temperature under air with monitoring by TLC. After 1 h, a solution of TFA (250 µL) in CH_2_Cl_2_ (250 µL, ACS grade) was added. After 1.5 h, the reaction was completed as determined by TLC analysis (silica, the product exhibited R_f_ = 0.63 with ethyl acetate). The reaction mixture was treated dropwise with saturated aqueous NaHCO_3_ solution. The organic layer was separated, dried (Na_2_SO_4_), and concentrated to a dark oil, which was passed through a short silica column (CH_2_Cl_2_/ethyl acetate (3:1)) to afford a colorless oil (36 mg, 99%): ^1^H-NMR (600 MHz, CDCl_3_) δ 3.73 (s, 3H), 3.81 (s, 2H), 6.65–6.66 (m, 1H), 6.80 (q, *J* = 2.5 Hz, 1H), 7.47–7.48 (m, 1H), 9.25 (bs, 1H); ^13^C{^1^H}-NMR (150 MHz, CDCl_3_) δ 46.8, 52.4, 109.0, 120.1, 124.5, 125.0, 168.6, 187.6; HRMS (ESI-TOF) *m/z*: [M + H]^+^ calcd. for C_8_H_10_NO_3_ 168.0655; found 168.0654.

#### 5.2.2. Route 2

*3-(3-Methoxy-1,3-dioxopropyl)pyrrole* (**3**). Following a reported procedure [3], a mixture of pyrrole **2-TIPS** (872 mg, 2.50 mmol), methyl potassium malonate (585 mg, 3.75 mmol), Xantphos (725 mg, 1.25 mmol), MgCl_2_ (357 mg, 3.75 mmol), and imidazole (330 mg, 4.85 mmol) was placed in a 50 mL Schlenk flask and charged with argon. THF (25.0 mL) was added followed by triethylamine (520 µL, 3.73 mmol). The mixture was degassed by three freeze–pump–thaw cycles. Then, Pd(OAc)_2_ (280 mg, 1.25 mmol) and Co_2_(CO)_8_ (430 mg, 1.20 mmol) were added. The flask was sealed immediately and heated at 65 °C in an oil bath for 48 h, and progress was monitored by removal of samples periodically followed by TLC analysis (silica, hexanes/ethyl acetate (1:1)). Upon completion, the reaction mixture was diluted with ethyl acetate and filtered through a Celite pad. The filtrate was washed with brine and water, dried (Na_2_SO_4_), and concentrated. The resulting residue was dissolved in ethyl acetate, passed through a short silica column, and concentrated to give a brown oil. The oil was treated with TBAF solution (1.0 M in THF, 2.7 mL) and stirred at room temperature for 2 h. The reaction mixture was diluted with ethyl acetate and washed with saturated aqueous NaHCO_3_ solution and brine. The organic phase was dried (Na_2_SO_4_) and concentrated. Purification by column chromatography (silica, CH_2_Cl_2_, then hexanes/ethyl acetate (1:1)) gave a brown oil (122 mg, 29%) with satisfactory characterization (^1^H-NMR and ESI-MS) data.

#### 5.2.3. Route 3

*3-(3-Methoxy-1,3-dioxopropyl)pyrrole* (**3**). Samples of **2-TIPS** (349 mg, 1.00 mmol), potassium monomethyl malonate (245 mg, 1.57 mmol), Xantphos (351 mg, 0.607 mmol), MgCl_2_ (149 mg, 1.56 mmol), and imidazole (134 mg, 1.97 mmol) were charged in a 50 mL Schlenk flask. Then, anhydrous THF (21 mL) and triethylamine (219 µL, 1.57 mmol) were sequentially added to the above mixture. The resulting mixture was degassed by three cycles of freeze–pump–thaw followed by loading of CO gas under ambient pressure and addition of Pd(OAc)_2_ (176 mg, 0.784 mmol). The reaction vessel was sealed, and the mixture was stirred in an oil bath at 65 °C for 48 h. The reaction mixture was diluted with ethyl acetate and filtered through a Celite pad. The filtrate was concentrated and purified by column chromatography (silica, hexanes/ethyl acetate (1:1 and then 1:2), 3 cm × 35 cm) to afford a yellow oil (72 mg, 43%): analysis by TLC (silica, *R*_f_ = 0.20 with hexanes/ethyl acetate (1:1)); ^1^H-NMR (500 MHz, CDCl_3_): δ 3.73 (s, 3H), 3.81 (s, 2H), 6.66–6.67 (m, 1H), 6.79–6.81 (m, 1H), 7.47–7.49 (m, 1H), 9.08 (br, 1H); ^13^C{^1^H}-NMR (125 MHz, CDCl_3_): δ 46.7, 52.4, 109.1, 120.0, 124.4, 125.1, 168.5, 187.5; HRMS (ESI-TOF) *m/z*: [M + H]^+^ calcd. for C_8_H_10_NO_3_, 168.0655; found, 168.0653.

#### 5.2.4. Route 4

*3-(3-Methoxy-1,3-dioxopropyl)pyrrole* (**3**). Example A: Following a reported procedure [21] with modification [4], methyl malonyl chloride (2.0 mL, 19 mmol) was added slowly to a stirred slurry of AlCl_3_ (2.4 g, 18 mmol) in 1,2-dichloroethane (25 mL) at 0 °C. After 20 min, **1-TIPS** (2.7 g, 12 mmol) was added dropwise. The mixture was stirred at 0 °C for 15 min, then for 3 h at room temperature. The mixture was poured into an ice-water mixture, which was then acidified by the addition of aqueous HCl solution (2 M). The resulting organic phase was separated and extracted with dichloromethane. The organic extract was washed with saturated aqueous NaHCO_3_ solution and brine, dried (Na_2_SO_4_), and concentrated. The resulting residue was treated with TBAF solution (1.0 M in THF, 12 mL) and stirred at room temperature for 2 h. The reaction mixture was diluted with ethyl acetate and then washed with saturated aqueous NaHCO_3_ solution and brine. The organic phase was dried (Na_2_SO_4_) and concentrated. Column chromatography (silica, hexanes/ethyl acetate (1:1)) gave the unwanted 2-substituted pyrrole isomer (275 mg, 14%) and the desired 3-substituted pyrrole title compound as a brown oil (309 mg, 15%): ^1^H-NMR (400 MHz) δ 3.73 (s, 3H), 3.81 (s, 2H), 6.66 (m, 1H), 6.80 (m, 1H), 7.48 (m, 1H), 9.13 (br, 1H); ^13^C{^1^H}-NMR (100 MHz) δ 46.4, 52.4, 108.7, 120.5, 124.7, 125.2, 168.8, 188.1; HRMS (ESI-MS) *m/z*: [M + Na]^+^ calcd. for C_8_H_9_NNaO_3_, 190.0475; found 190.0480.

Example B: Following a reported procedure [21] with modification [4], methyl malonyl chloride (16.0 mL, 150 mmol) was added dropwise to a suspension of AlCl_3_ (20.0 g, 150 mmol) in anhydrous 1,2-dichloroethane (200 mL) at 0 °C. After 15 min, **1-TIPS** (22.34 g, 100 mmol) was added dropwise to the reaction mixture at 0 °C. The resulting mixture was stirred for 3 h at room temperature, then quenched by pouring the reaction contents into a beaker containing ice water. The mixture was then concentrated under reduced pressure to remove 1,2-dichloroethane (which otherwise gave an emulsion upon addition of ethyl acetate and attempted aqueous extraction) followed by treatment with aqueous HCl solution (2 M, 200 mL). The resulting mixture was extracted with ethyl acetate (3 × 150 mL). The combined organic extract was washed with saturated aqueous NaHCO_3_ (2 × 200 mL) and saturated brine (1 × 100 mL), dried (Na_2_SO_4_), and concentrated. The resulting brown oil was then treated with TBAF solution (1.0 M in THF, 150 mL) and stirred for 1 h at room temperature. The reaction mixture was treated with saturated aqueous NaHCO_3_ (200 mL). The aqueous layer was extracted with CH_2_Cl_2_ (3 × 150 mL). The combined organic extract was washed with brine (1 × 100 mL), dried (Na_2_SO_4_), and concentrated. The crude product was purified by column chromatography (silica, column size 8 cm × 20 cm), hexanes/ethyl acetate (1:1)) to obtain the unwanted isomer (**4**) followed by the title compound (**3**).

Data for 3-(3-methoxy-1,3-dioxopropyl)pyrrole (**3**): 1.10 g, 7%; analysis by TLC (silica, R_f_ = 0.21 with hexanes/ethyl acetate (1:1)) as a brown oil. ^1^H-NMR (500 MHz, CDCl_3_) δ 3.72 (s, 3H), 3.80 (s, 2H), 6.64−6.65 (m, 1H), 6.78−6.79 (m, 1H), 7.46−7.47 (m, 1H), 9.22 (bs, 1H); ^13^C{^1^H}-NMR (126 MHz, CDCl_3_) δ 46.8, 52.5, 109.1, 120.2, 124.6, 125.1, 168.6, 187.6; HRMS *m/z*: [M + H]^+^ calcd. for C_8_H_10_NO_3_, 168.06552, found 168.06541; HRMS *m/z*: [M + Na]^+^ calcd. for C_8_H_9_NNaO_3_, 190.04746, found 190.04736.

Data for 2-(3-methoxy-1,3-dioxopropyl)pyrrole (**4**): 6.16 g, 36%; analysis by TLC (silica, R_f_ = 0.42 with hexanes/ethyl acetate (1:1)) as a red oil. ^1^H-NMR (500 MHz, CDCl_3_) δ 3.73 (s, 3H), 3.81 (s, 2H), 6.29−6.30 (m, 1H), 6.95−6.96 (m, 1H), 7.09−7.10 (m, 1H), 9.82 (bs, 1H); ^13^C{^1^H}-NMR (150 MHz, CDCl_3_) δ 45.0, 52.6, 111.3, 118.1, 126.2, 131.3, 168.1, 181.8; HRMS *m/z*: [M + H]^+^ calcd. for C_8_H_10_NO_3_, 168.06552, found 168.06541; HRMS *m/z*: [M + Na]^+^ calcd. for C_8_H_9_NNaO_3_, 190.04746, found 190.04724.

#### 5.2.5. Exploratory Acylation Studies

*2-(3-Methoxy-1,3-dioxopropyl)pyrrole* (**4**). Following a reported procedure [21] with modification [4], methyl malonyl chloride (800 μL, 7.5 mmol) was added dropwise to a suspension of AlCl_3_ (1.0 g, 7.5 mmol) in anhydrous 1,2-dichloroethane (10 mL) at 0 °C. After 10 min, pyrrole (350 μL, 5.0 mmol) was added dropwise to the reaction mixture at 0 °C. The resulting mixture was stirred for 3 h at room temperature, then quenched by pouring the reaction contents into a beaker containing ice water. The mixture was then concentrated under reduced pressure to remove 1,2-dichloroethane (which otherwise gave an emulsion upon addition of ethyl acetate and attempted aqueous extraction) followed by treatment with aqueous HCl solution (2 M, 50 mL). The resulting mixture was extracted with ethyl acetate (3 × 50 mL). The combined organic extract was washed with saturated aqueous NaHCO_3_ (2 × 100 mL) and saturated brine (1 × 50 mL), dried (Na_2_SO_4_), and concentrated. The crude product was purified by column chromatography [silica, hexanes/ethyl acetate (1:1)] to obtain a yellow oil (246 mg, 30%). The ^1^H and ^13^C{^1^H}-NMR data were consistent with those reported above.

*3-(3-Methoxy-1,3-dioxopropyl)-N-triisopropylpyrrole* (**3-TIPS**)**.** Example using pyridine: Following a reported procedure [21] with modification, pyridine (610 µL, 7.6 mmol) was added dropwise to a solution of methyl malonyl chloride (650 µL, 6.1 mmol) in anhydrous 1,2-dichloroethane (22 mL) at 0 °C. After stirring for 15 min, **1-TIPS** (1.2 mL, 4.9 mmol) was added dropwise to the reaction mixture at 0 °C. The resulting solution was refluxed for 24 h, then quenched by the addition of saturated aqueous NH_4_Cl solution (50 mL). The aqueous layer was extracted by CH_2_Cl_2_ (3 × 20 mL). The organic extract was washed with saturated aqueous NaHCO_3_ (2 × 30 mL) and saturated brine (1 × 30 mL), dried (Na_2_SO_4_), and concentrated. The crude product was purified by column chromatography (silica, hexanes/ethyl acetate (100:0 then 5:1)) to obtain two unknown products.

Survey of Lewis acids: Following a reported procedure [29], a sample of a Lewis acid (0.1 mmol) was added to a stirred solution of **1-TIPS** (240 µL, 1.0 mmol) and methyl malonyl chloride (160 µL, 1.5 mmol) in nitromethane (1.0 mL). The reaction was monitored by TLC analysis. After stirring for 5 h at room temperature, the reaction was quenched by treatment with saturated aqueous NaHCO_3_ (5.0 mL). The aqueous layer was extracted with CH_2_Cl_2_ (3 × 5 mL). The combined organic extract was dried (Na_2_SO_4_) and concentrated to obtain a viscous dark crude oil. The ^1^H-NMR spectrum was identical with that of the 2-acylated product **4** reported above.

Example using AlCl_3_: Following a reported procedure [21] with modification [4], methyl malonyl chloride (800 μL, 7.5 mmol) was added dropwise to a suspension of AlCl_3_ (1.0 g, 7.5 mmol) in anhydrous 1,2-dichloroethane (10 mL) at 0 °C. After 10 min, **1-TIPS** (1.2 mL, 4.9 mmol) was added dropwise to the reaction mixture at 0 °C. The resulting mixture was stirred for 3 h at room temperature, then quenched by pouring the reaction contents into a beaker containing ice water. The mixture was then concentrated under reduced pressure to remove 1,2-dichloroethane (which otherwise gave an emulsion upon addition of ethyl acetate and attempted aqueous extraction) followed by treatment with aqueous HCl solution (2 M, 50 mL). The resulting mixture was extracted with ethyl acetate (3 × 50 mL). The combined organic extract was washed with saturated aqueous NaHCO_3_ (2 × 100 mL) and saturated brine (1 × 50 mL), dried (Na_2_SO_4_), and concentrated. The crude product was purified by column chromatography (silica, hexanes/ethyl acetate (30:1 then 5:1 then 1:1)) to obtain **3-TIPS** (157 mg, 10% as a black solid) followed by **4** (143 mg, 17% as a black oil). The ^1^H and ^13^C{^1^H}-NMR data were consistent with those reported above for each compound.

#### 5.2.6. Intermediate for XRD Study

*3-(3-Methoxy-1,3-dioxopropyl)-N-triisopropylpyrrole* (**3-TIPS**). Following a general procedure [3], a mixture of **2-TIPS** (349 mg, 1.00 mmol), potassium 3-methoxy-3-oxopropanoate (245 mg, 1.57 mmol), Xantphos (351 mg, 0.607 mmol), MgCl_2_ (149 mg, 1.56 mmol), and imidazole (134 mg, 1.97 mmol) was charged in a 50 mL Schlenk flask. Then, anhydrous THF (21 mL) and triethylamine (219 µL, 1.57 mmol) were sequentially added. The resulting mixture was degassed by three cycles of freeze–pump–thaw followed by loading of argon gas. Pd(OAc)_2_ (176 mg, 0.784 mmol) and Co_2_(CO)_8_ (284 mg, 0.79 mmol) were added. A brisk evolution of gas was observed during the course of addition. The flask was then capped with a rubber septum fitted with a needle connected to an empty balloon for venting the excess gas. The reaction mixture was heated at 65 °C for 48 h. The reaction mixture was diluted with ethyl acetate and filtered through a Celite pad. The filtrate was concentrated and purified by column chromatography (silica, hexanes/ethyl acetate (30:1 and then 5:1), 5 cm × 26 cm) to furnish a brown solid (103 mg, 32%): analysis by TLC (silica, *R*_f_ = 0.35 in hexanes/ethyl acetate (5:1)); m.p. 76–78 °C; ^1^H-NMR (500 MHz, CDCl_3_): δ 1.10 (d, *J* = 7.6 Hz, 18H), 1.47 (hept, *J* = 7.5 Hz, 3H), 3.73 (s, 3H), 3.80 (s, 2H), 6.71 (dd, *J* = 2.9 and 1.4 Hz, 1H), 6.73–6.74 (m, 1H), 7.45 (d, *J* = 1.7 Hz, 1H); ^13^C{^1^H}-NMR (125 MHz, CDCl_3_): δ 11.5, 17.6, 47.1, 52.3, 111.0, 125.8, 127.2, 130.5, 168.5, 187.1; HRMS (ESI-TOF) *m/z*: [M + H]^+^ calcd. for C_17_H_30_NO_3_Si, 324.1990; found, 324.1980.

#### 5.2.7. XRD Analysis

A sample of **3-TIPS** was crystallized by slow evaporation of a solution of CDCl_3_ from an NMR tube over some days. A resulting colorless block-like specimen of C_17_H_29_NO_3_Si, approximate dimensions 0.154 × 0.204 × 0.254 mm, was used for the X-ray crystallographic analysis. The X-ray intensity data were measured on a Bruker-Nonius X8 Kappa APEX II system equipped with a fine-focus sealed tube (MoKα, λ = 0.71073 Å) and a graphite monochromator. The total exposure time was 3.88 h. The frames were integrated with the Bruker SAINT software package using a narrow-frame algorithm. The integration of the data using an orthorhombic unit cell yielded a total of 64,836 reflections to a maximum θ angle of 30.99° (0.69 Å resolution), of which 5920 were independent (average redundancy 10.952, completeness = 100.0%, R_int_ = 6.33%, R_sig_ = 3.14%) and 4552 (76.89%) were greater than 2σ(F^2^). The final cell constants of a = 7.9903(3) Å, b = 14.9649(7) Å, c = 31.0291(13) Å, volume = 3710.3(3) Å^3^ are based upon the refinement of the XYZ-centroids of 2055 reflections above 20 σ(I) with 5.222° < 2θ < 68.33°. Data were corrected for absorption effects using the Multi-Scan method (SADABS). The ratio of minimum to maximum apparent transmission was 0.952. The calculated minimum and maximum transmission coefficients (based on crystal size) were 0.9660 and 0.9790.

The structure was solved and refined using the Bruker SHELXTL Software Package, using the space group P b c a, with Z = 8 for the formula unit, C_17_H_29_NO_3_Si. The final anisotropic full-matrix least-squares refinement on F^2^ with 206 variables converged at R_1_ = 3.73% for the observed data and wR_2_ = 9.99% for all data. The goodness-of-fit was 1.030. The largest peak in the final difference electron density synthesis was 0.456 e^-^/Å^3^, and the largest hole was -0.221 e^-^/Å^3^ with an RMS deviation of 0.053 e^-^/Å^3^. On the basis of the final model, the calculated density was 1.158 g/cm^3^ and F(000), 1408 e^−^.

These structural data for **3-TIPS** (CCDC 2226334) can be obtained free of charge via https://www.ccdc.cam.ac.uk/structures/Search?Ccdcid=2226334&DatabaseToSearch=Published (or from the CCDC, 12 Union Road, Cambridge CB2 1EZ, UK; Fax: +44 1223 336033; E-mail: deposit@ccdc.cam.ac.uk), accessed on 28 January 2023.

## 6. Outlook

The full synthesis of any native bacteriochlorophylls or chlorophylls has not yet been achieved [1]. The syntheses reported herein provide straightforward access to a precursor of an analogue of native ring C. The scope (>5 mmol) and efficacy (one day at most) from commercially available TIPS-pyrrole (**1-TIPS**) via route 4 are suitable to meet the stated objectives for incorporation in a planned program for the synthesis of photosynthetic tetrapyrrole macrocycles and analogues. Still, improved reaction conditions remain an important focus to streamline synthetic access more fully in this domain, illustrated by the multiple products (and the unexplained, variable ratios in batch-to-batch runs) obtained in the Friedel–Crafts acylation of **1-TIPS.** Access to precursors to ring C and analogues may also prove useful in the synthesis of phyllobilins [42,43]—catabolic products of chlorophyll that contain an intact ring E—for which general syntheses also remain to be developed.

## Figures and Tables

**Table 1:** Spectral parameters of native versus naked macrocycles *^a^*.

	Absorption	B_x_ Band	B_y_ Band	Q_x_ Band	Q_y_ Band
Compound		λ_abs_ (fwhm), ε	λ_abs_ (fwhm), ε	λ_abs_ (fwhm), ε	λ_abs_ (fwhm), ε
**Bacteriopheophytin *a***		356 (46), ^*b*,*c*^ 115,000	384 (23), ^*b*,*c*^ 74,700	525 (24), 36,400	750 (37), 71,000
**H_2_BC**		340 (12), ^*b*,*d*^ 112,000	365 (14), *^d^* 130,000	489 (10), 33,000	713 (12), 112,000
**Pheophytin *a***		407 (54), *^e^* 112,000		– *^f^*	667 (17.5), 55,100
**H_2_C**		389 (33), *^e^* 158,000		– *^f^*	634 (9), 66,500

*^a^* Units of λ_abs_ and fwhm are in nm; units of ε are in M^−1^cm^−1^. *^b^* Assessed by a Gaussian fit. *^c^* Combined B_x_ and B_y_ bands have fwhm = 58.5 nm. *^d^* Combined B_x_ and B_y_ bands have fwhm = 36 nm. *^e^* The B_x_ and B_y_ bands are coalesced. *^f^* See spectra.

## Data Availability

X-ray crystal data are deposited at CCDC for **3-TIPS** (2226334). All other data are contained within the paper and Supplementary Materials.

## Supplementary Materials

The following supporting information can be downloaded at https://www.mdpi.com/article/10.3390/molecules28031323/s1. Table S1: Single-crystal X-ray structure data for **3-TIPS**; ^1^H-NMR and ^13^C{^1^H}-NMR data for all new compounds, comprising 25 pages.

## Sample Availability

Samples have generally been consumed during the course of research.
