# Supplementary material for: Four Routes to 3-(3-Methoxy-1,3-dioxopropyl)pyrrole, a Core Motif of Rings C and E in Photosynthetic Tetrapyrroles
Source: Molecules. 2023 Jan 30;28(3):1323. doi: 10.3390/molecules28031323 (PMC9920783; doi:10.3390/molecules28031323)
Supplement: Supplementary file 1 [file molecules-28-01323-s001.zip › molecules-2154378-supplementary.pdf]

Supplementary Materials for:

Four Routes to 3-(3-Methoxy-1,3-dioxopropyl)pyrrole, a Core Motif of Rings C and E in Photosynthetic Tetrapyrroles

Khiem Chau Nguyen,[a] Anh Thu Nguyen Tran,[a] Pengzhi Wang,[a] Shaofei Zhang, Zhiyuan Wu, and Jonathan S. Lindsey*

[a]Equal contributions by three authors

Department of Chemistry
North Carolina State University
Raleigh, NC 27695-8204
e-mail: jlindsey@ncsu.edu

Table of Contents

	Topic	Page
(1)	Single-crystal X-ray diffraction data	S2
(2)	NMR spectra	S3–S25

Table S1. Single-crystal X-ray structure data for **3-TIPS**

CCDC registry	2226334
Chemical formula	$C_{17}H_{29}NO_3Si$
Formula weight (g/mol)	323.50
Temperature (K)	100 (2)
Wavelength (Å)	0.71073
Crystal size (mm)	0.254 × 0.204 × 0.154
Crystal habit	Colorless block
Crystal system	Orthorhombic
Space group	*P* b c a
Unit cell dimensions, *a* (Å)	7.9903 (3)
Unit cell dimensions, *b* (Å)	14.9649 (7)
Unit cell dimensions, *c* (Å)	31.0291 (13)
α, degree	90
β, degree	90
γ, degree	90
Volume ($Å^3$)	3710.3 (3)
Z	8
Density (calculated), g/cm^3	1.158
F(000)	1408
Absorption coefficient, mm^{-1}	0.138
θ range for data collection, degree	2.63–30.99
Index ranges	-11<=h<=11, -21<=k<=21, -44<=l<=44
Reflections collected	64836
Independent reflections	5920 [R(int) = 0.0633]
R_1	0.0373
wR_2	0.0889
R_1 (all data)	0.0566
wR_2 (all data)	0.0999
Largest diff. peak and hole ($eÅ^{-3}$)	0.456 and -0.221
R.M.S. deviation from mean ($eÅ^{-3}$)	0.053

^1H NMR ($CDCl_3$, 600 MHz)

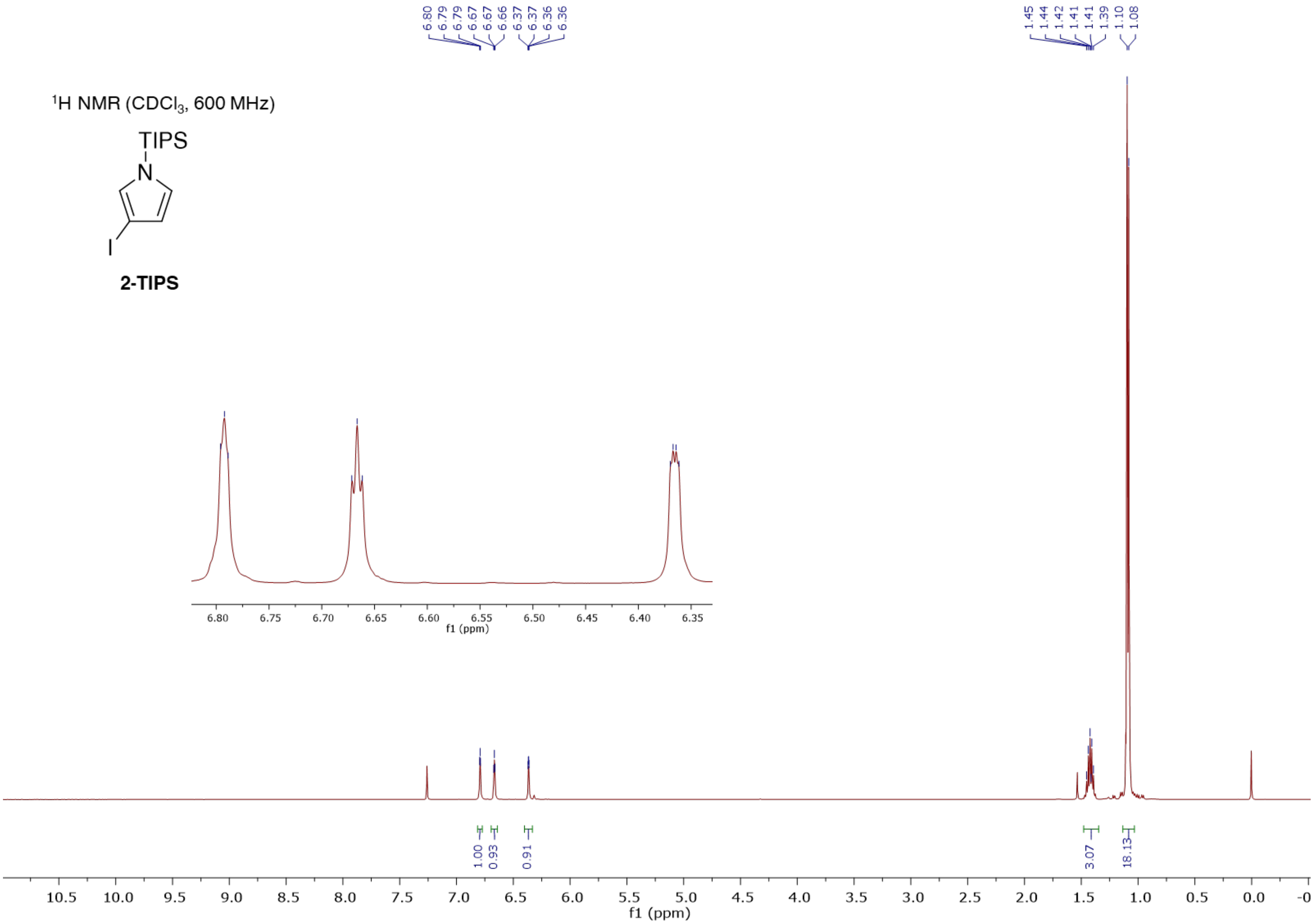

2-TIPS

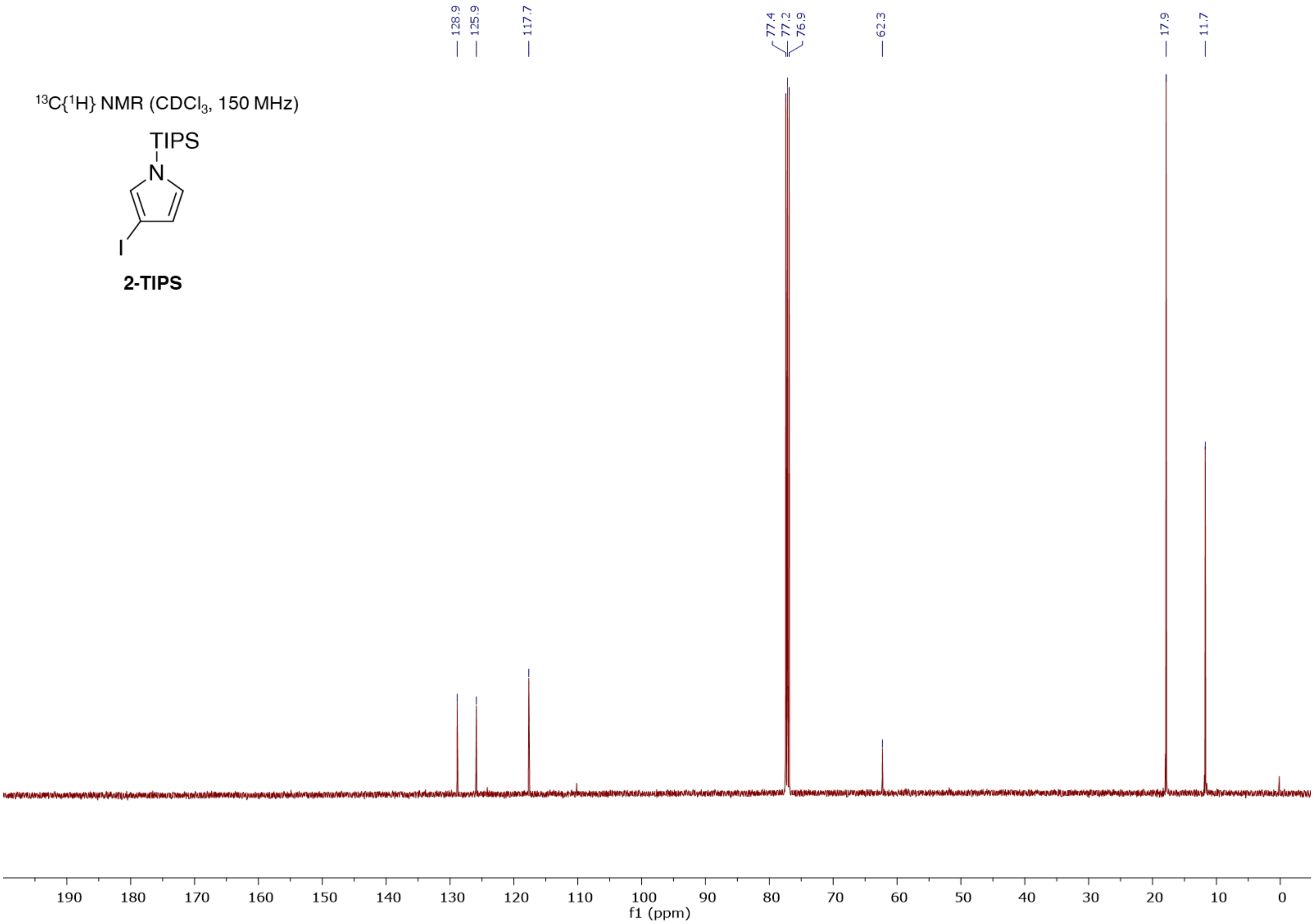

128.9
125.9
117.7
77.4
77.2
76.9
62.3
17.9
11.7
$^{13}C\{^1H\}$ NMR ($CDCl_3$, 150 MHz)
TIPS
N
I
2-TIPS
190
180
170
160
150
140
130
120
110
100
90
80
70
60
50
40
30
20
10
0
f1 (ppm)

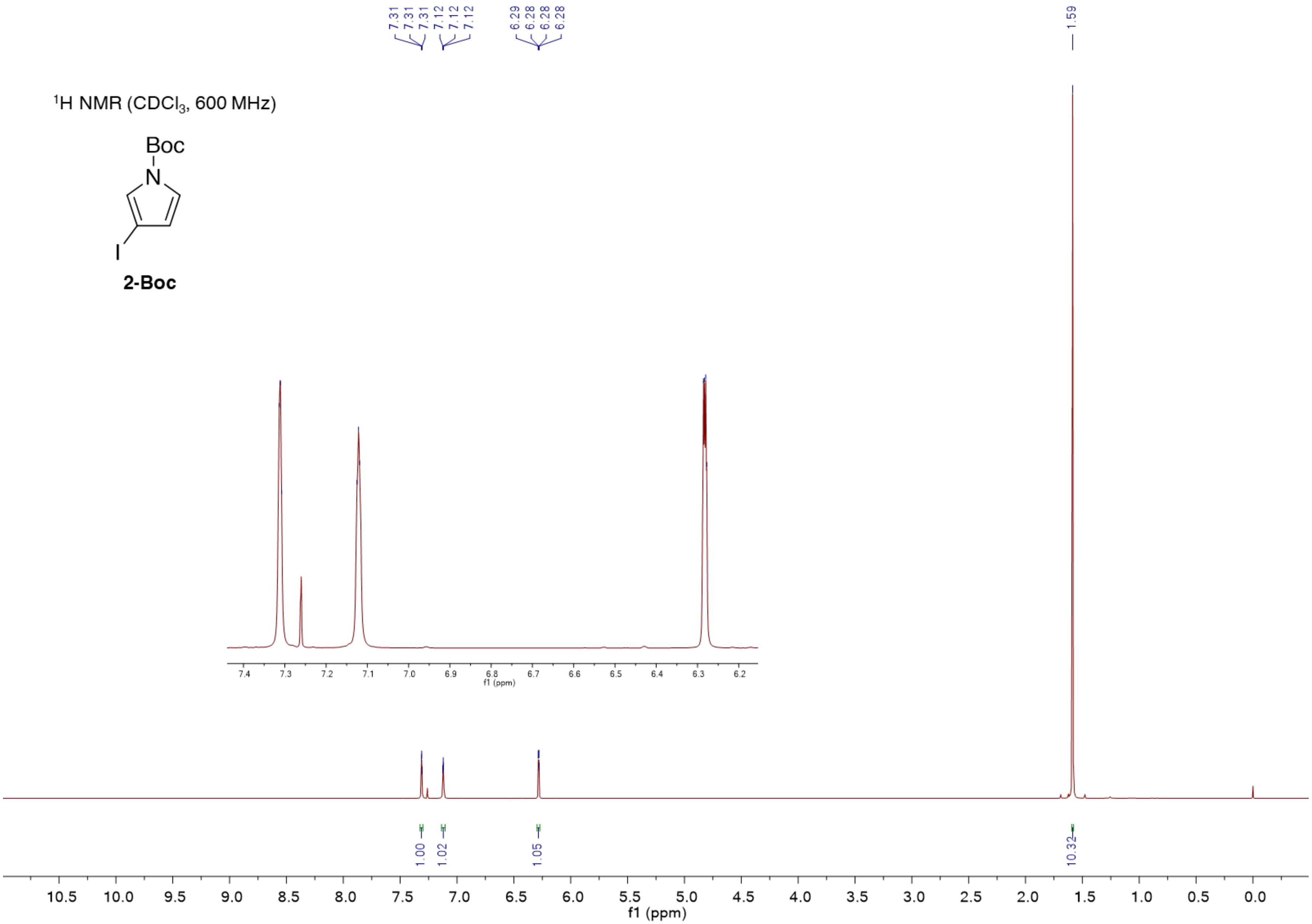

7.31
7.31
7.31
7.12
7.12
7.12
6.29
6.28
6.28
6.28
1.59
^{1}H NMR ($CDCl_3$, 600 MHz)
Boc
N
I
2-Boc
1.00
1.02
1.05
10.32
10.5 10.0 9.5 9.0 8.5 8.0 7.5 7.0 6.5 6.0 5.5 5.0 4.5 4.0 3.5 3.0 2.5 2.0 1.5 1.0 0.5 0.0
f1 (ppm)

$^{13}C\{^1H\}$ NMR ($CDCl_3$, 150 MHz)

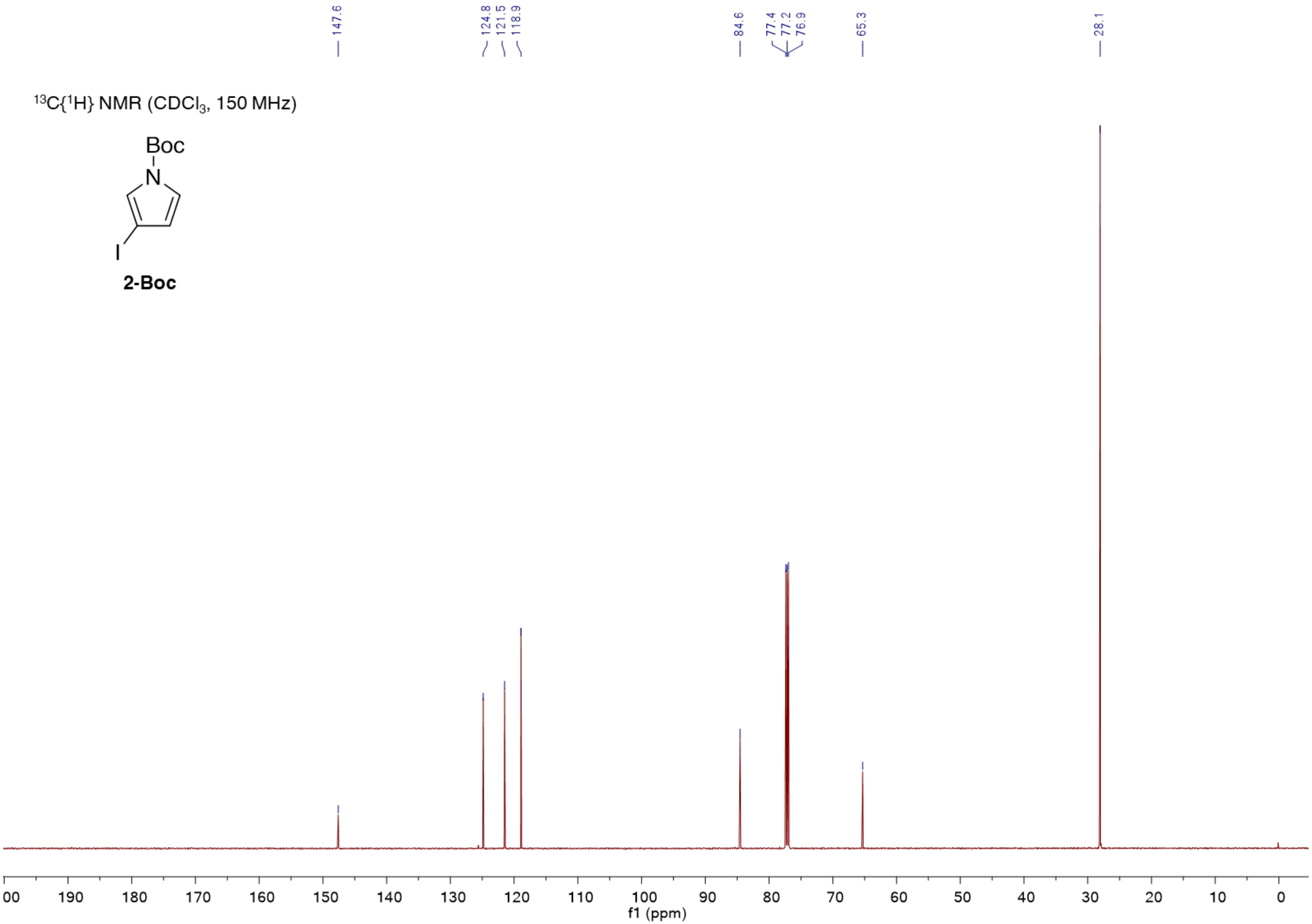

2-Boc

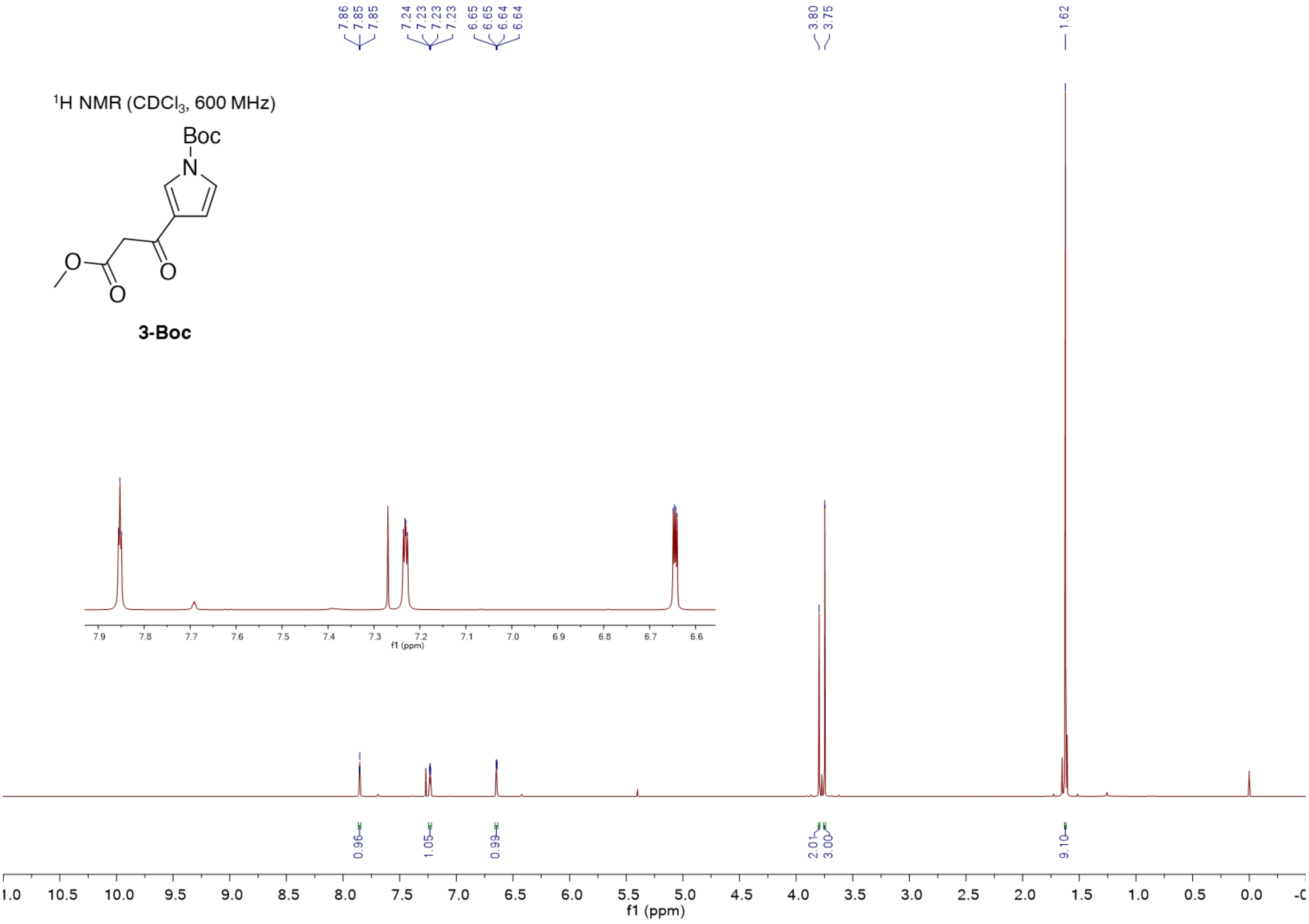
1H NMR (CDCl3, 600 MHz)
Boc
N
O
O
O
3-Boc
7.86
7.85
7.85
7.24
7.23
7.23
7.23
6.65
6.65
6.64
6.64
3.80
3.75
1.62
0.96
1.05
0.99
2.01
3.00
9.10
f1 (ppm)

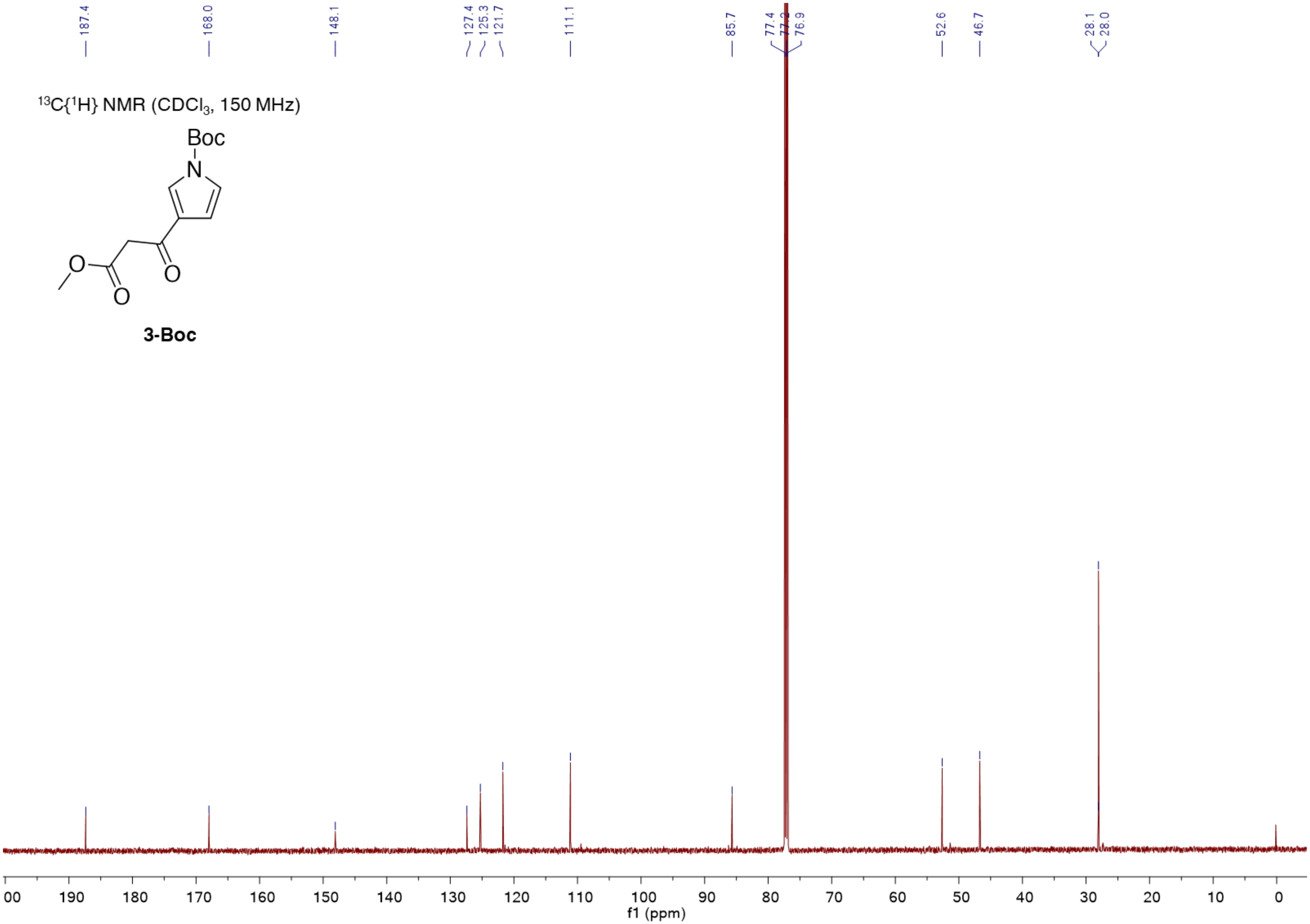
187.4
168.0
148.1
127.4
125.3
121.7
111.1
85.7
77.4
76.9
52.6
46.7
28.1
28.0
13C{1H} NMR (CDCl3, 150 MHz)
Boc
N
O
O
O
3-Boc
00
190
180
170
160
150
140
130
120
110
100
90
80
70
60
50
40
30
20
10
0
f1 (ppm)

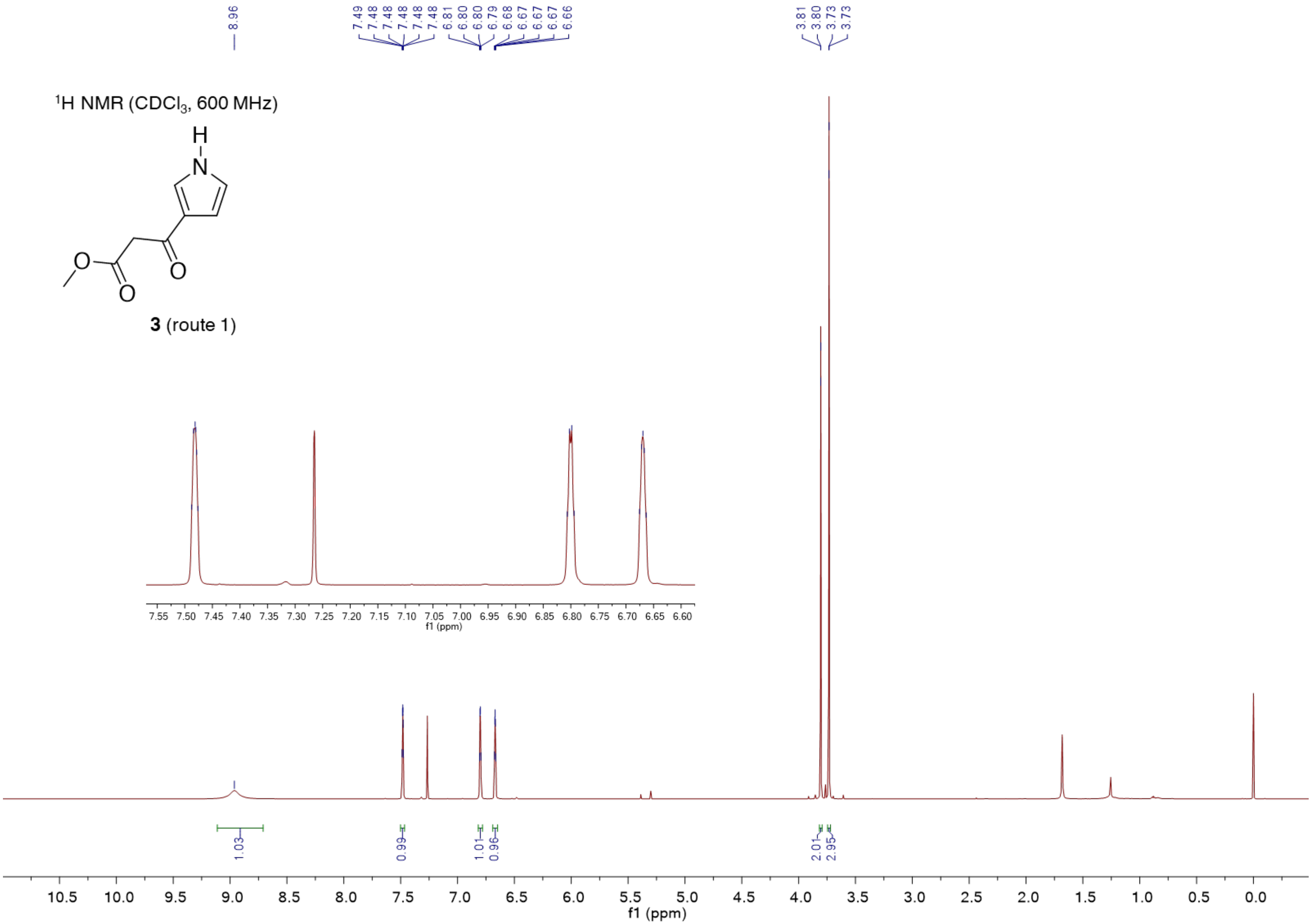

8.96
7.49 7.48 7.48 7.48 7.48 7.48 6.81 6.80 6.80 6.79 6.68 6.67 6.67 6.67 6.66
3.81 3.80 3.73 3.73
^{1}H NMR ($CDCl_3$, 600 MHz)
3 (route 1)
7.55 7.50 7.45 7.40 7.35 7.30 7.25 7.20 7.15 7.10 7.05 7.00 6.95 6.90 6.85 6.80 6.75 6.70 6.65 6.60
f1 (ppm)
1.03 0.99 1.01 0.96 2.01 2.95
10.5 10.0 9.5 9.0 8.5 8.0 7.5 7.0 6.5 6.0 5.5 5.0 4.5 4.0 3.5 3.0 2.5 2.0 1.5 1.0 0.5 0.0
f1 (ppm)

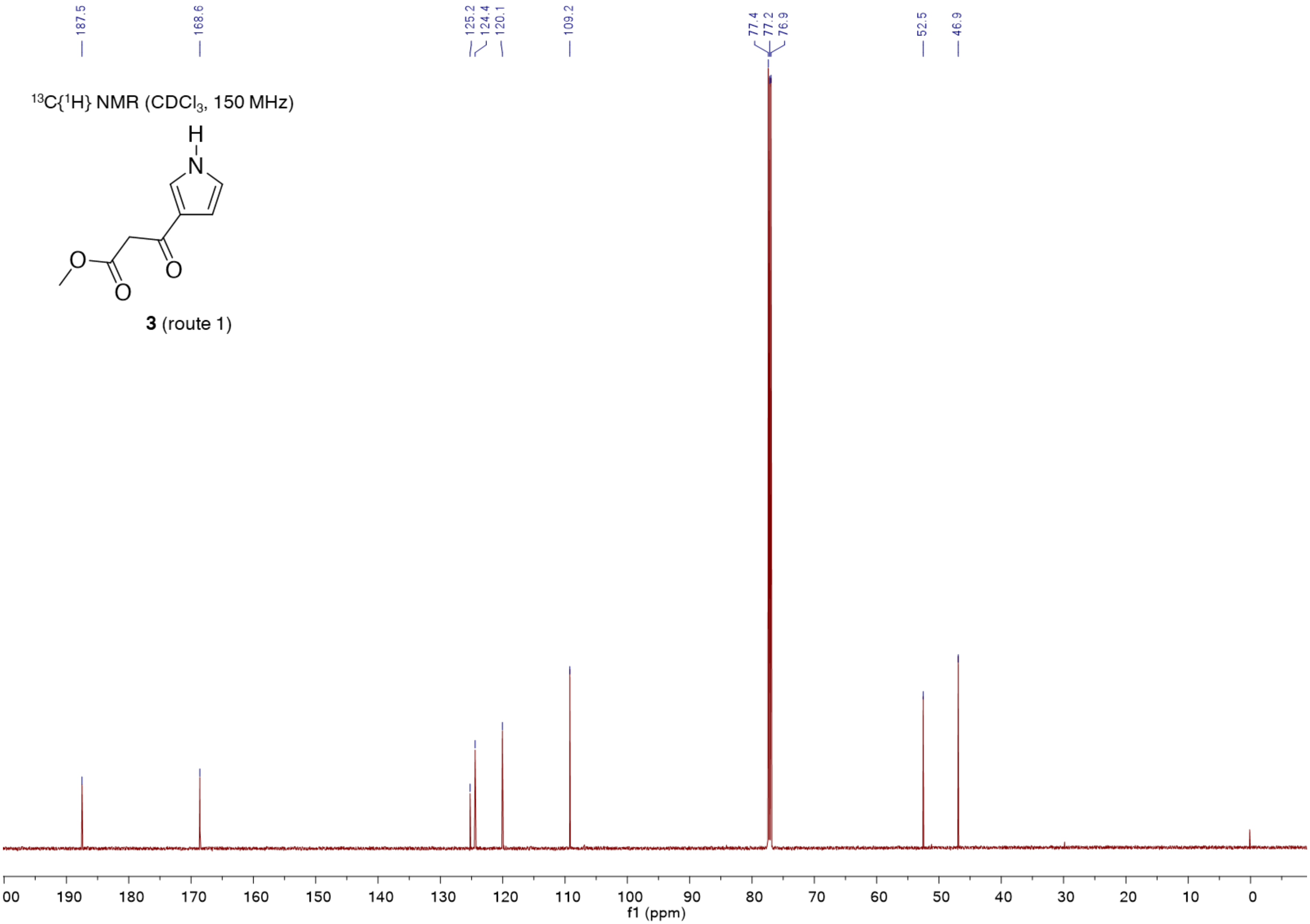
187.5
168.6
125.2
124.4
120.1
109.2
77.4
77.2
76.9
52.5
46.9
13C{1H} NMR (CDCl3, 150 MHz)
3 (route 1)
00 190 180 170 160 150 140 130 120 110 100 90 80 70 60 50 40 30 20 10 0
f1 (ppm)

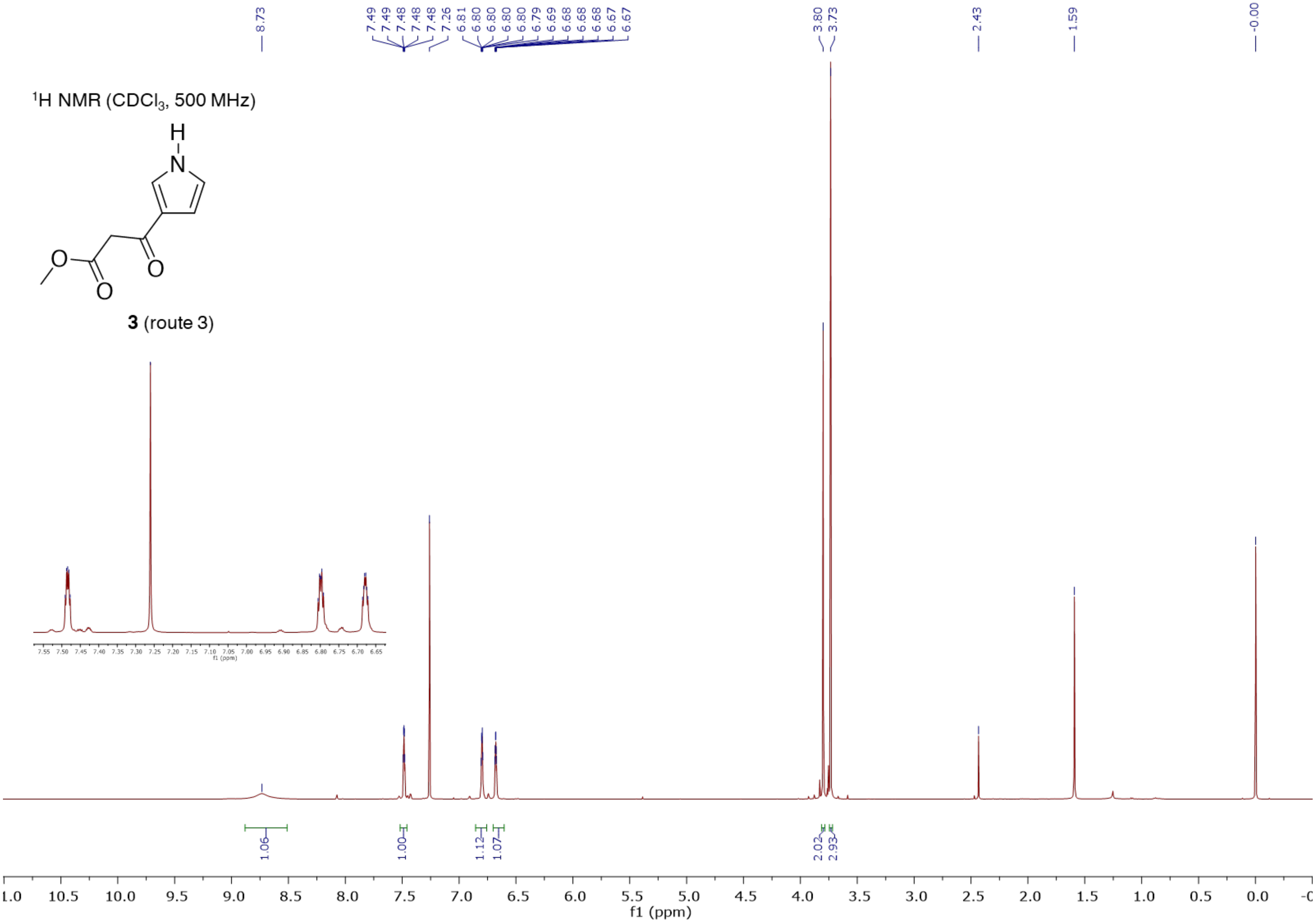

^{1}H NMR ($CDCl_3$, 500 MHz)

3 (route 3)

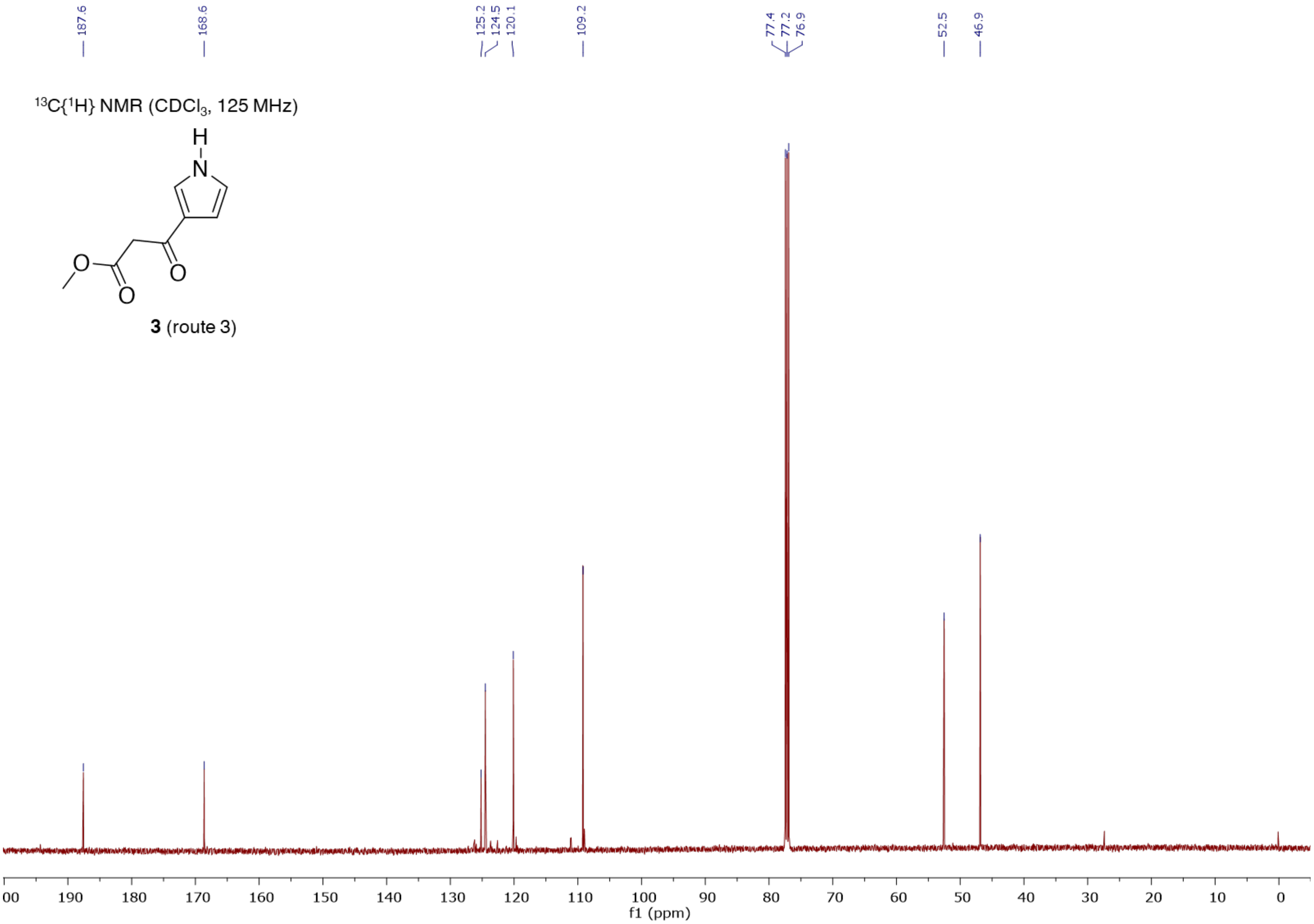

$^{13}C\{^1H\}$ NMR ($CDCl_3$, 125 MHz)

3 (route 3)

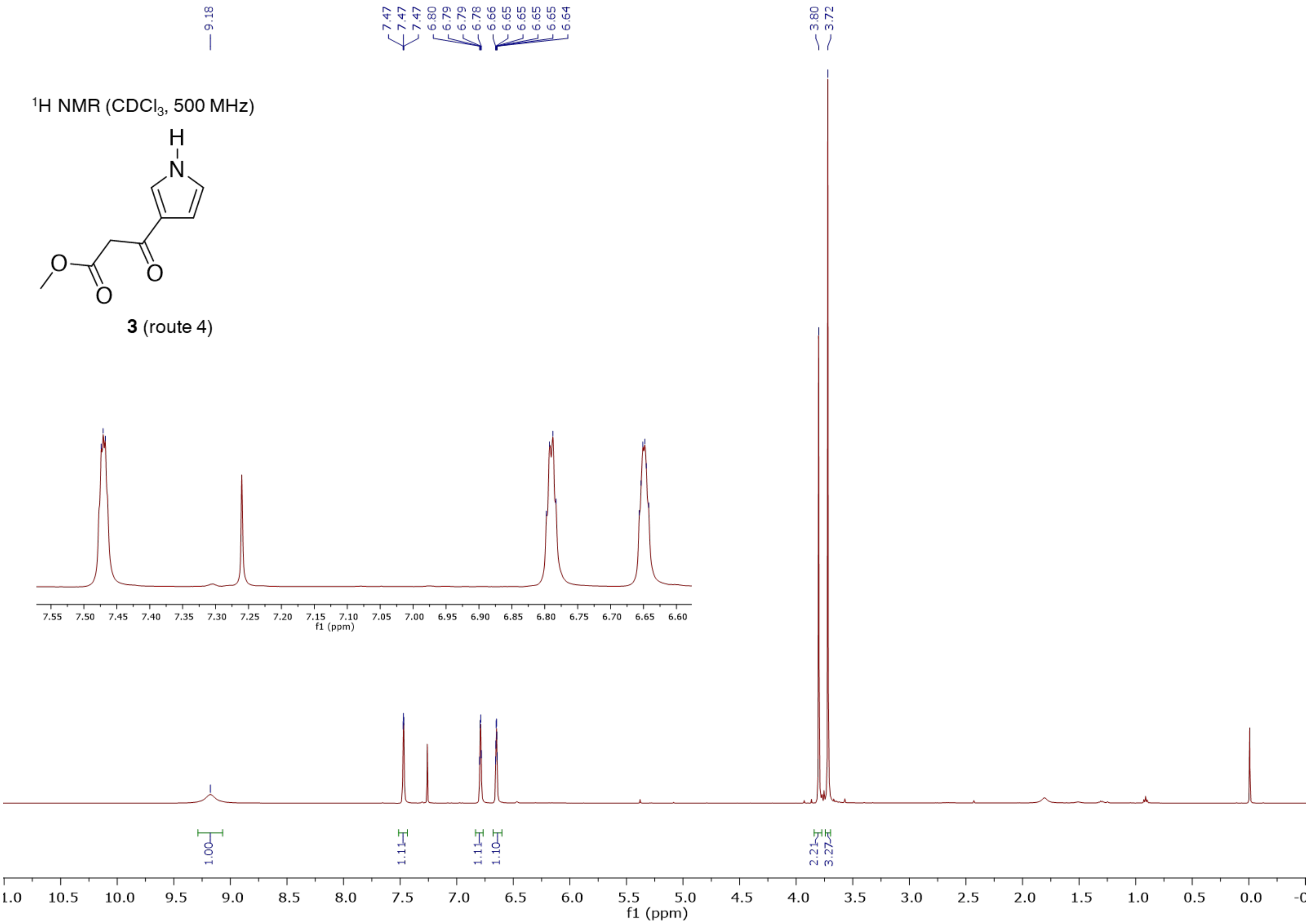

9.18
7.47
7.47
7.47
6.80
6.79
6.79
6.78
6.66
6.65
6.65
6.65
6.65
6.64
3.80
3.72
1H NMR (CDCl3, 500 MHz)
H
N
O
O
O
3 (route 4)
1.00
1.11
1.11
1.10
2.21
3.27
f1 (ppm)
f1 (ppm)

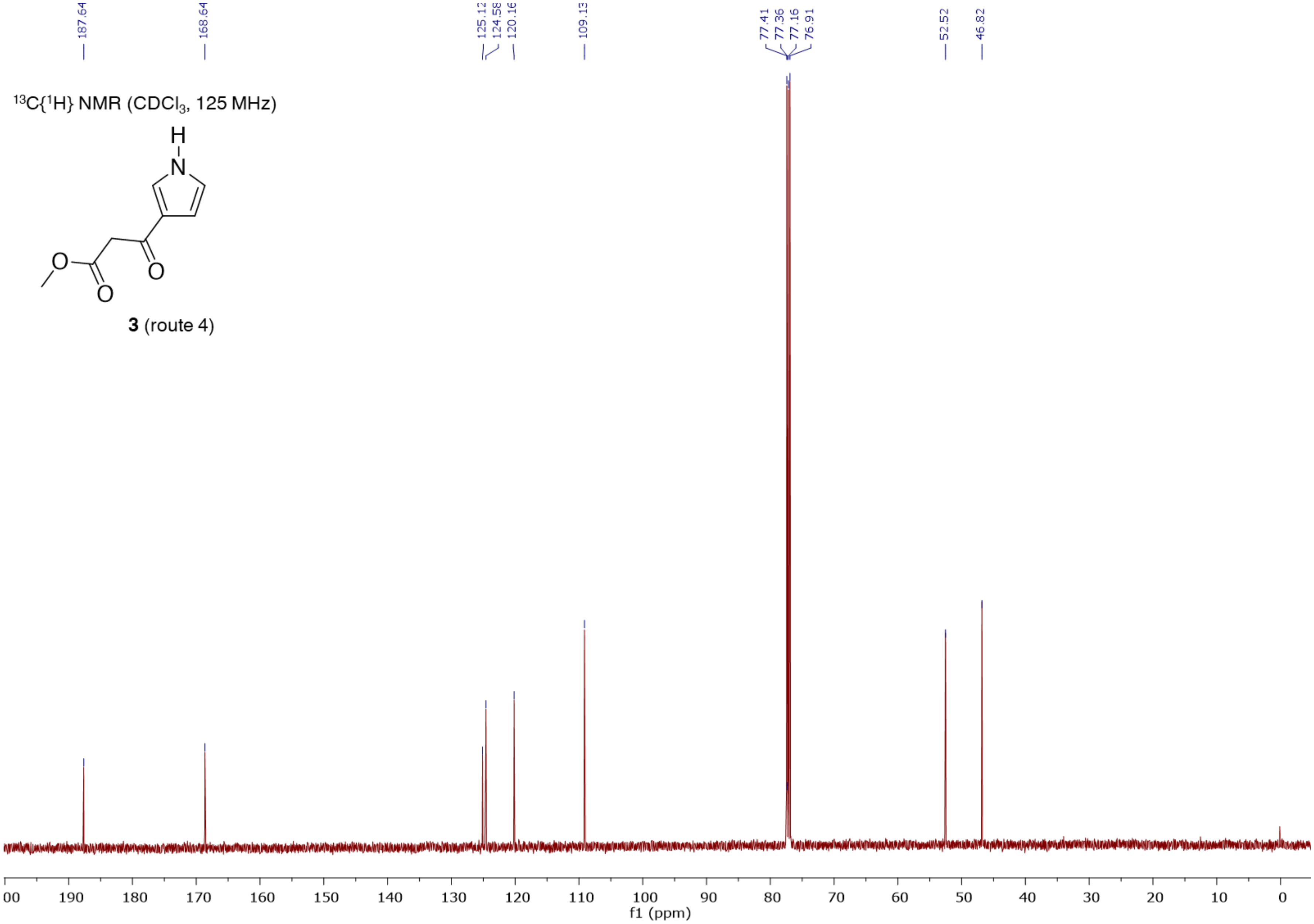
187.64
168.64
125.12
124.58
120.16
109.13
77.41
77.36
77.16
76.91
52.52
46.82
$^{13}C\{^{1}H\}$ NMR ($CDCl_3$, 125 MHz)
H
N
O
O
O
3 (route 4)
00
190
180
170
160
150
140
130
120
110
100
90
80
70
60
50
40
30
20
10
0
f1 (ppm)

COSY ($CDCl_3$, 500 MHz)

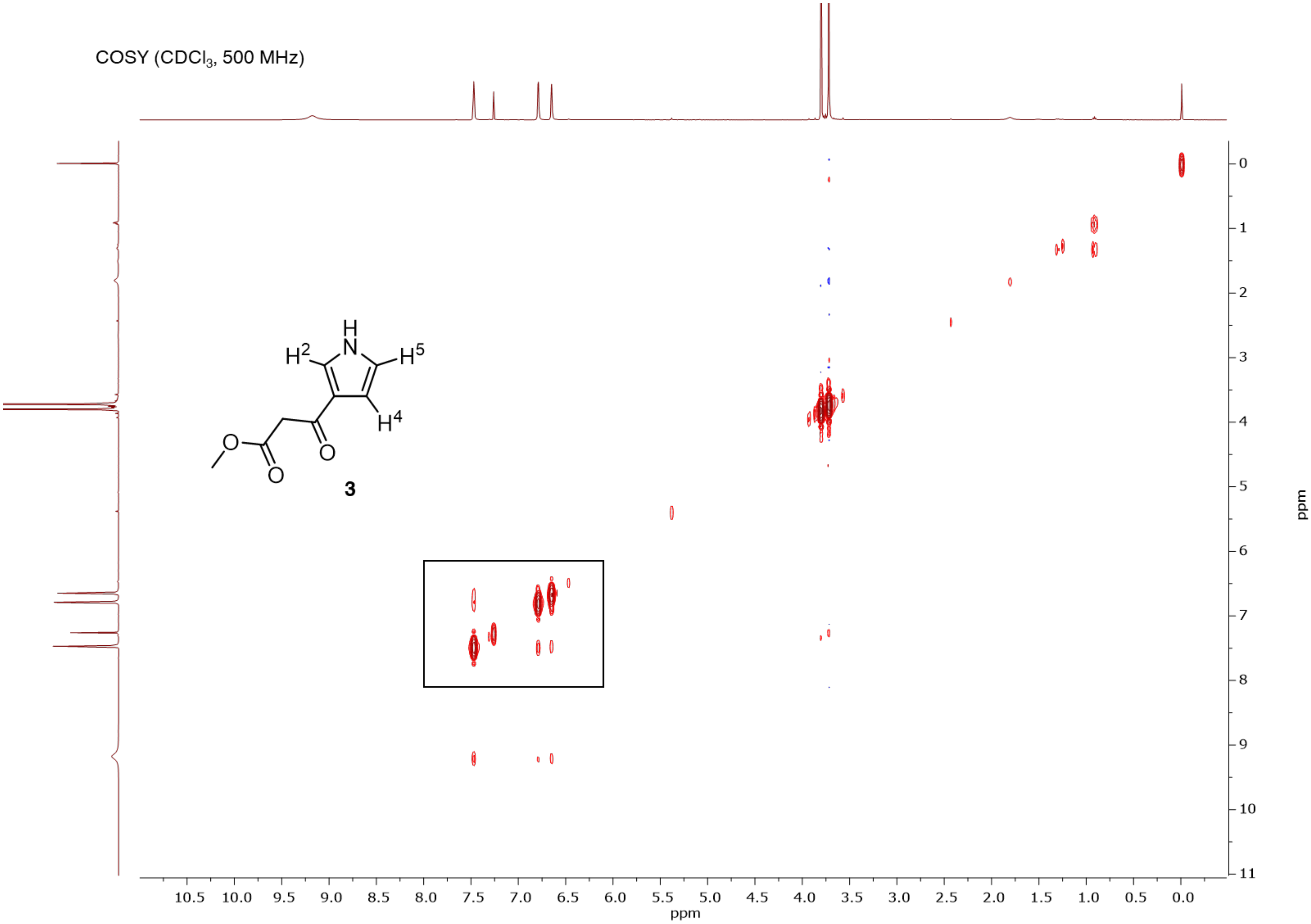

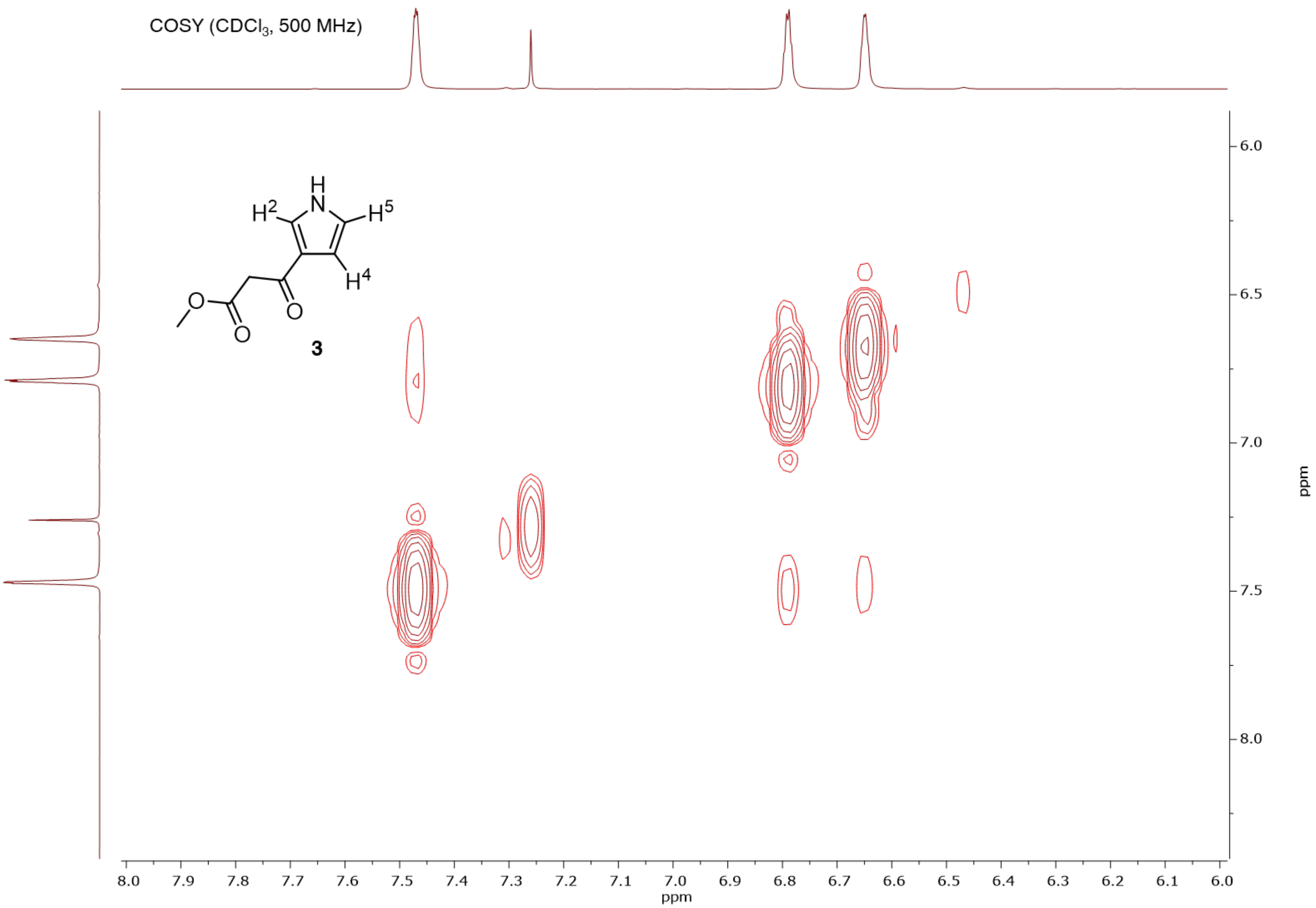
COSY (CDCl$_3$, 500 MHz)
H2
H
N
H5
H4
O
O
O
3
ppm
ppm

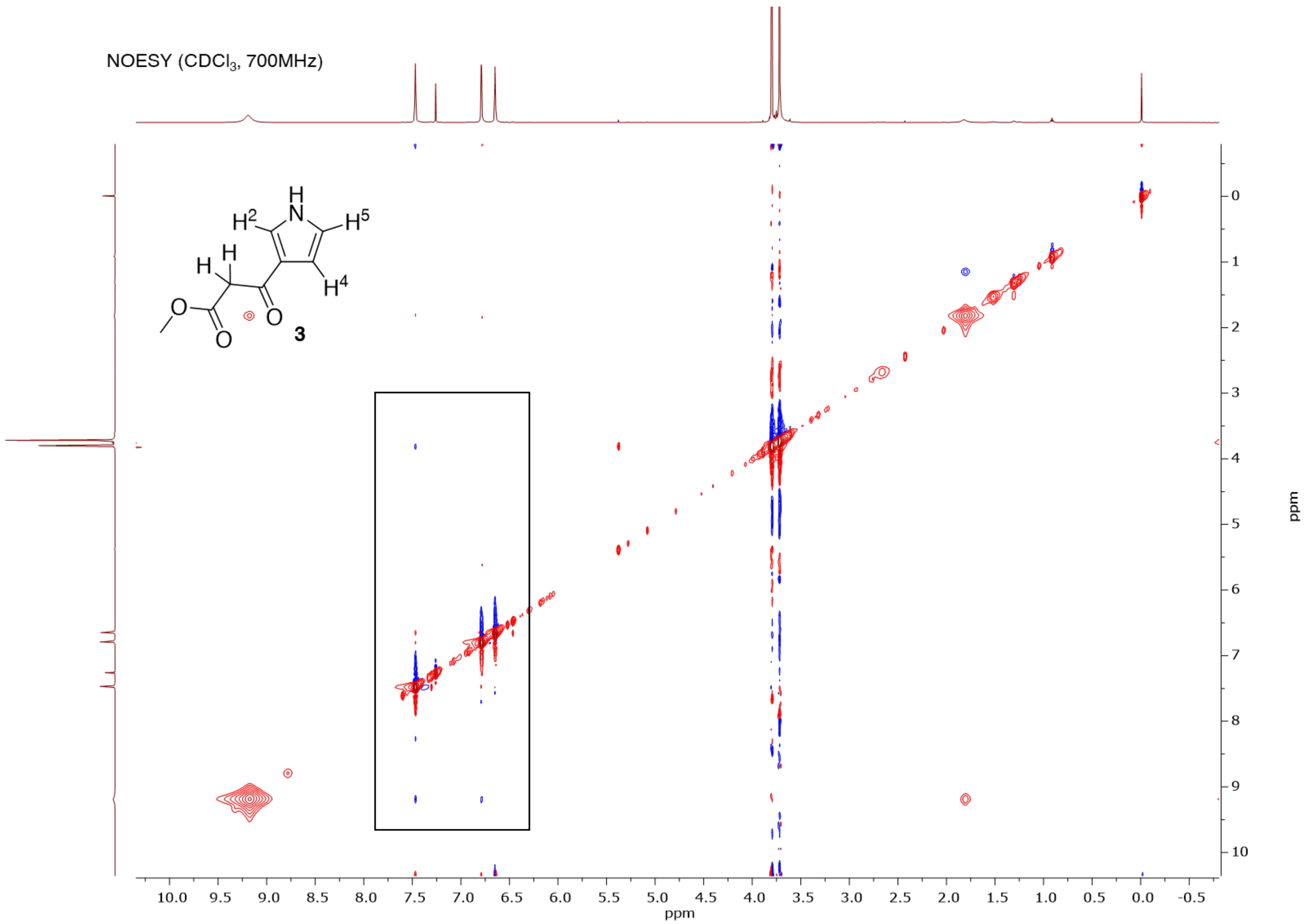
NOESY (CDCl$_3$, 700MHz)
H
N
H^2
H^5
H^4
H
H
O
O
O
3
10.0 9.5 9.0 8.5 8.0 7.5 7.0 6.5 6.0 5.5 5.0 4.5 4.0 3.5 3.0 2.5 2.0 1.5 1.0 0.5 0.0 -0.5
ppm
0 1 2 3 4 5 6 7 8 9 10
ppm

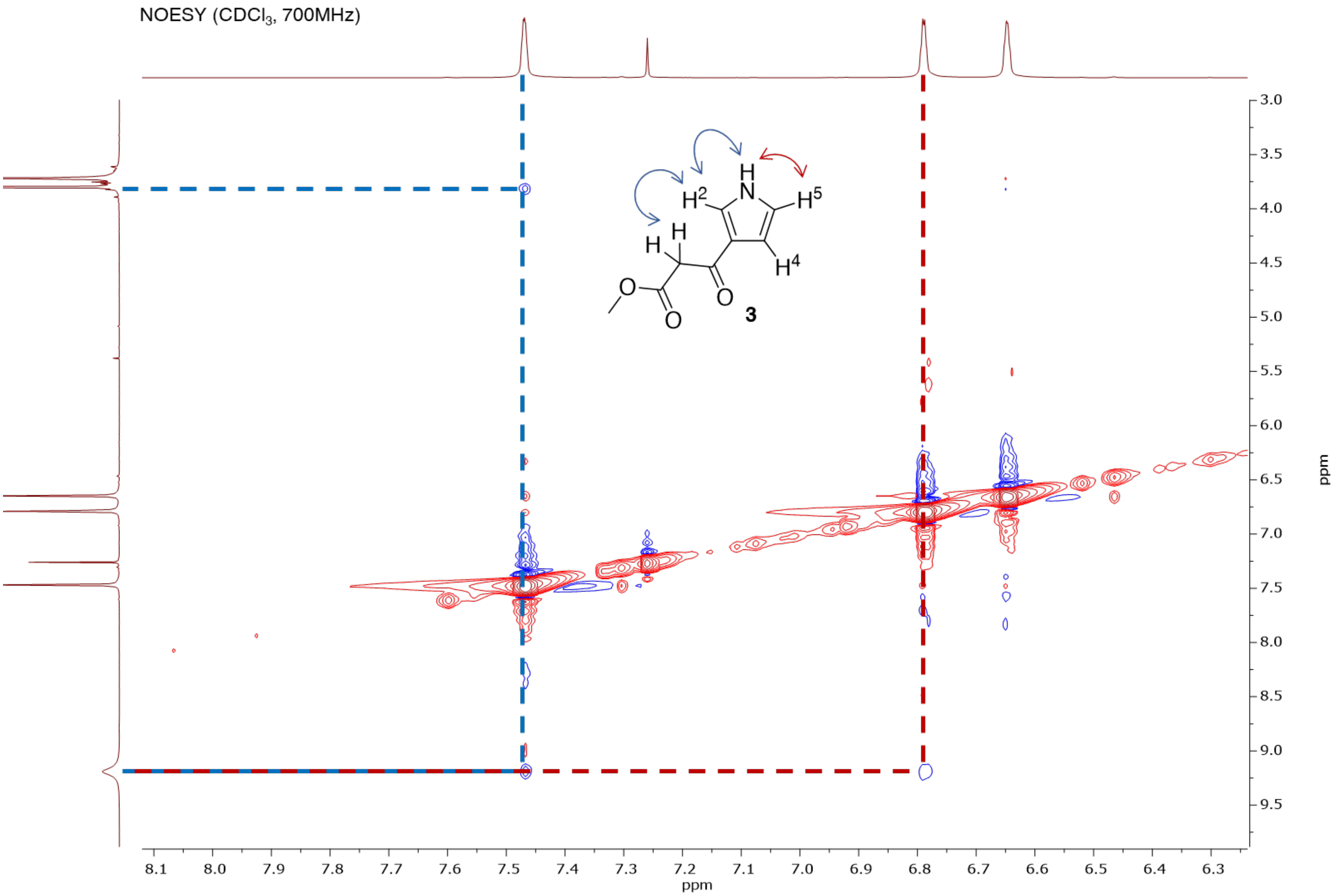
NOESY (CDCl$_3$, 700MHz)
H
N
H^2
H^5
H
H
H^4
O
O
O
3
ppm
ppm

^1H NMR ($CDCl_3$, 500 MHz)

4

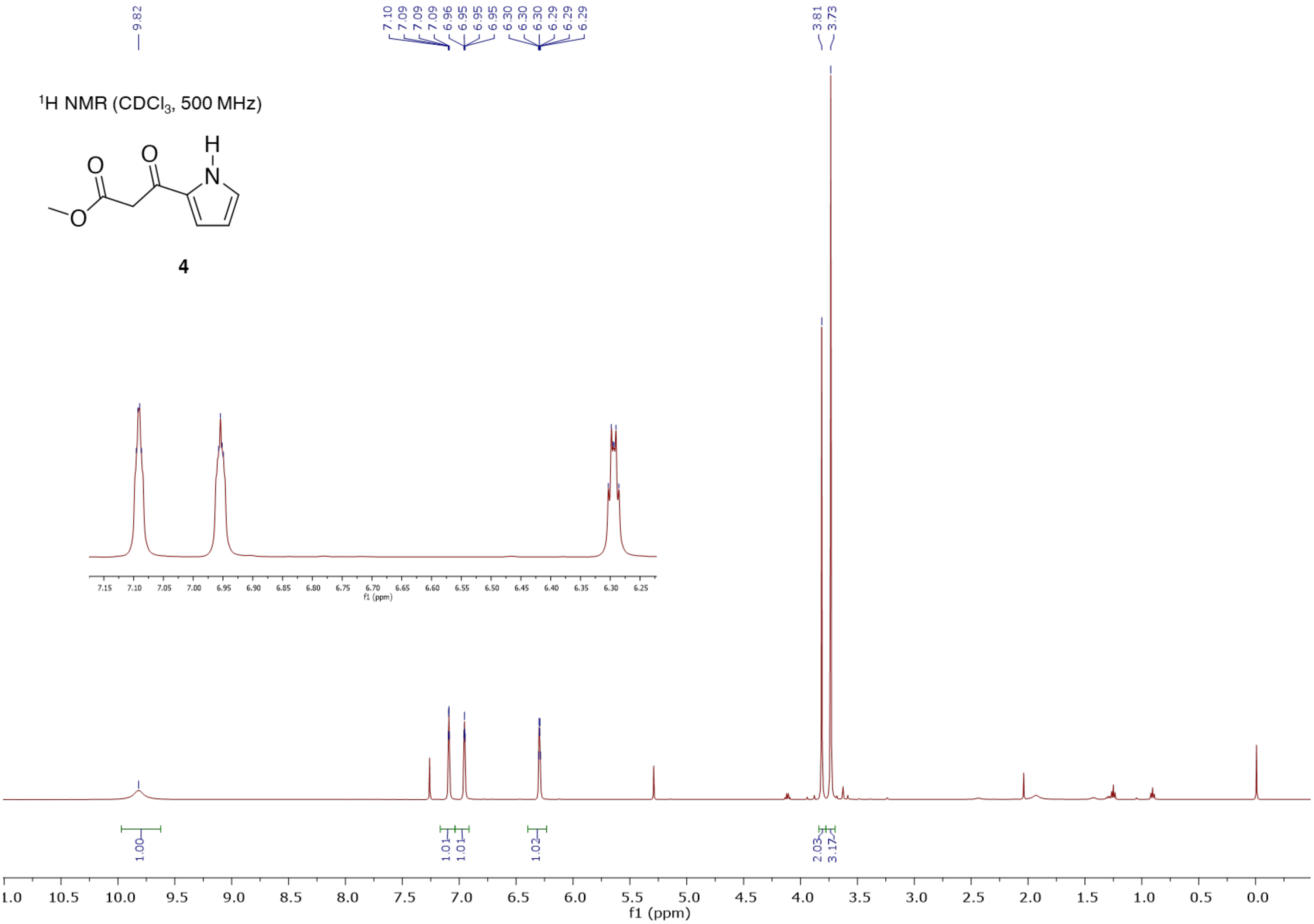

$^{13}C\{^{1}H\}$ NMR ($CDCl_3$, 125 MHz)

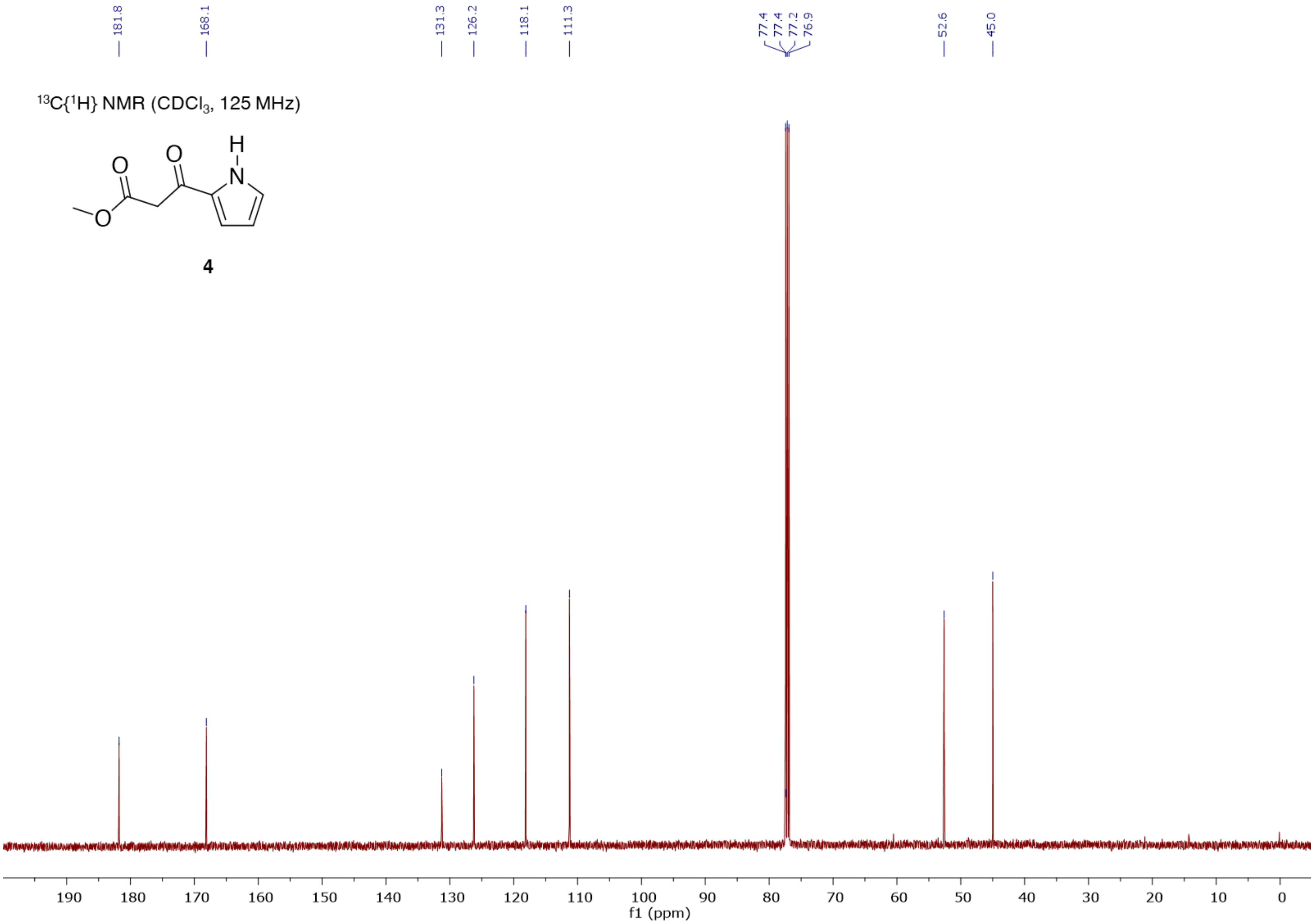

4

COSY ($CDCl_3$, 500 MHz)

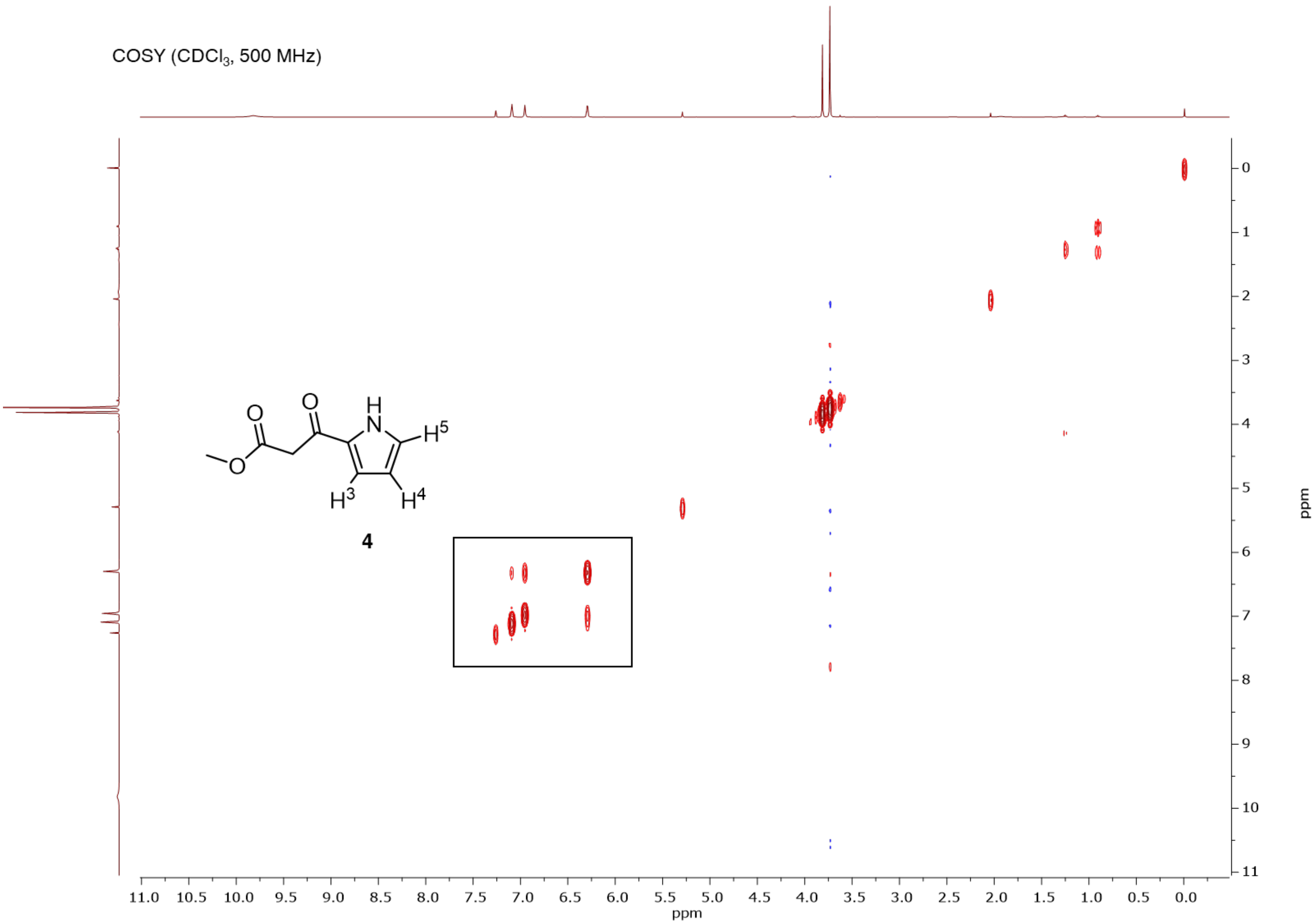

4

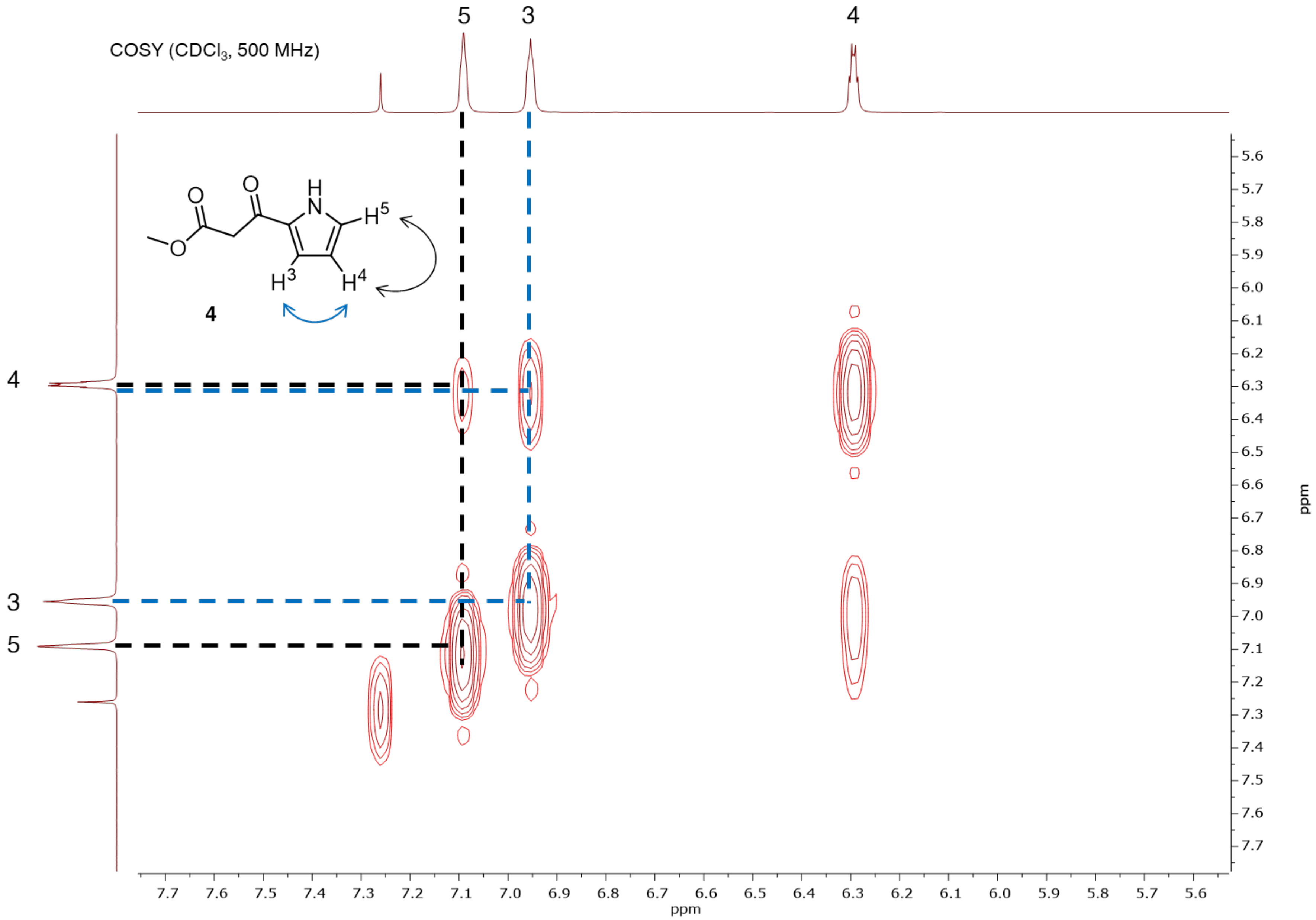

COSY (CDCl3, 500 MHz)
5
3
4
H
N
O
O
O
H5
H3
H4
4
4
3
5
ppm
ppm

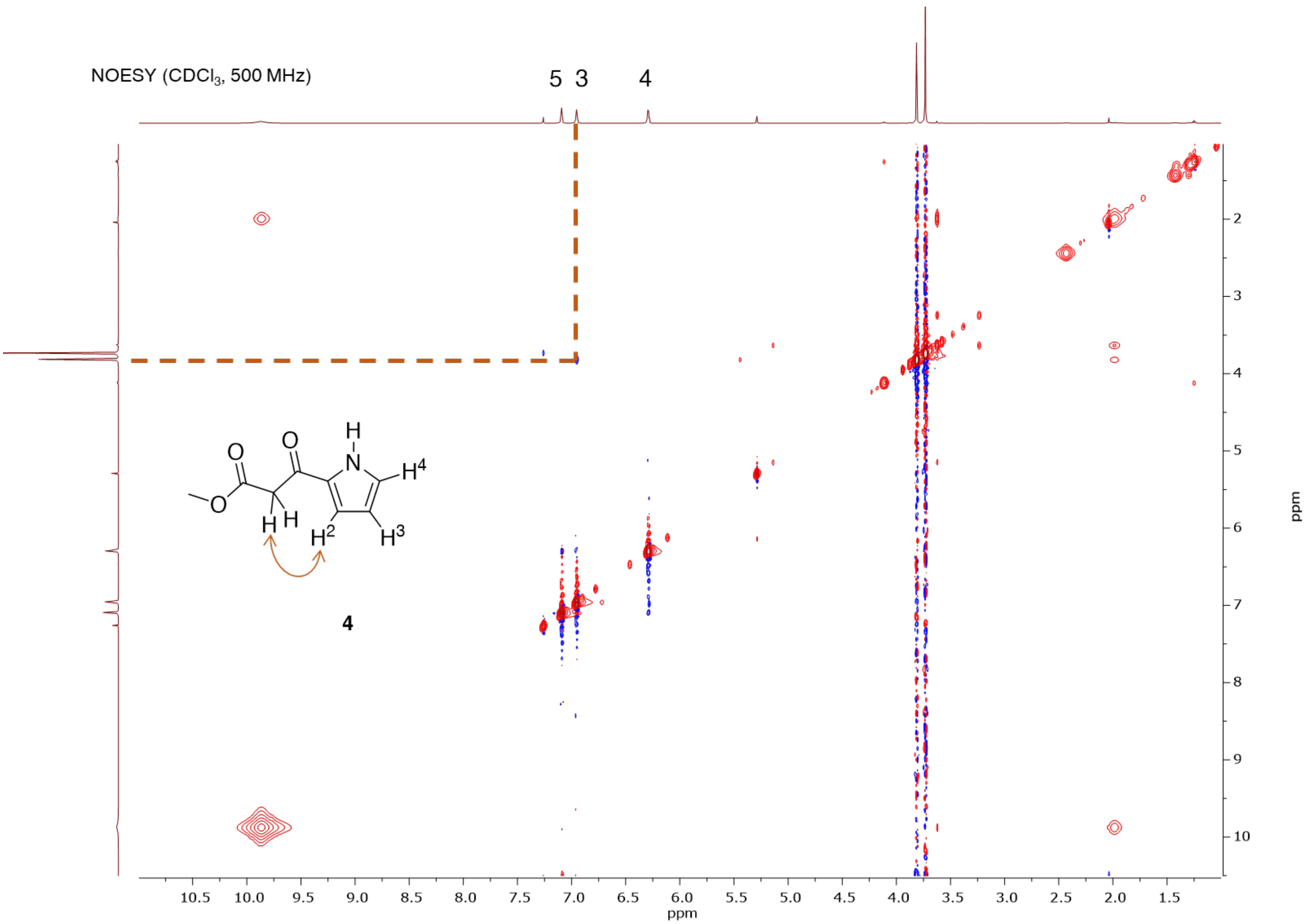
NOESY (CDCl3, 500 MHz)
5
3
4
H
N
H4
O
O
O
H
H
H2
H3
4
10.5
10.0
9.5
9.0
8.5
8.0
7.5
7.0
6.5
6.0
5.5
5.0
4.5
4.0
3.5
3.0
2.5
2.0
1.5
ppm
2
3
4
5
6
7
8
9
10
ppm

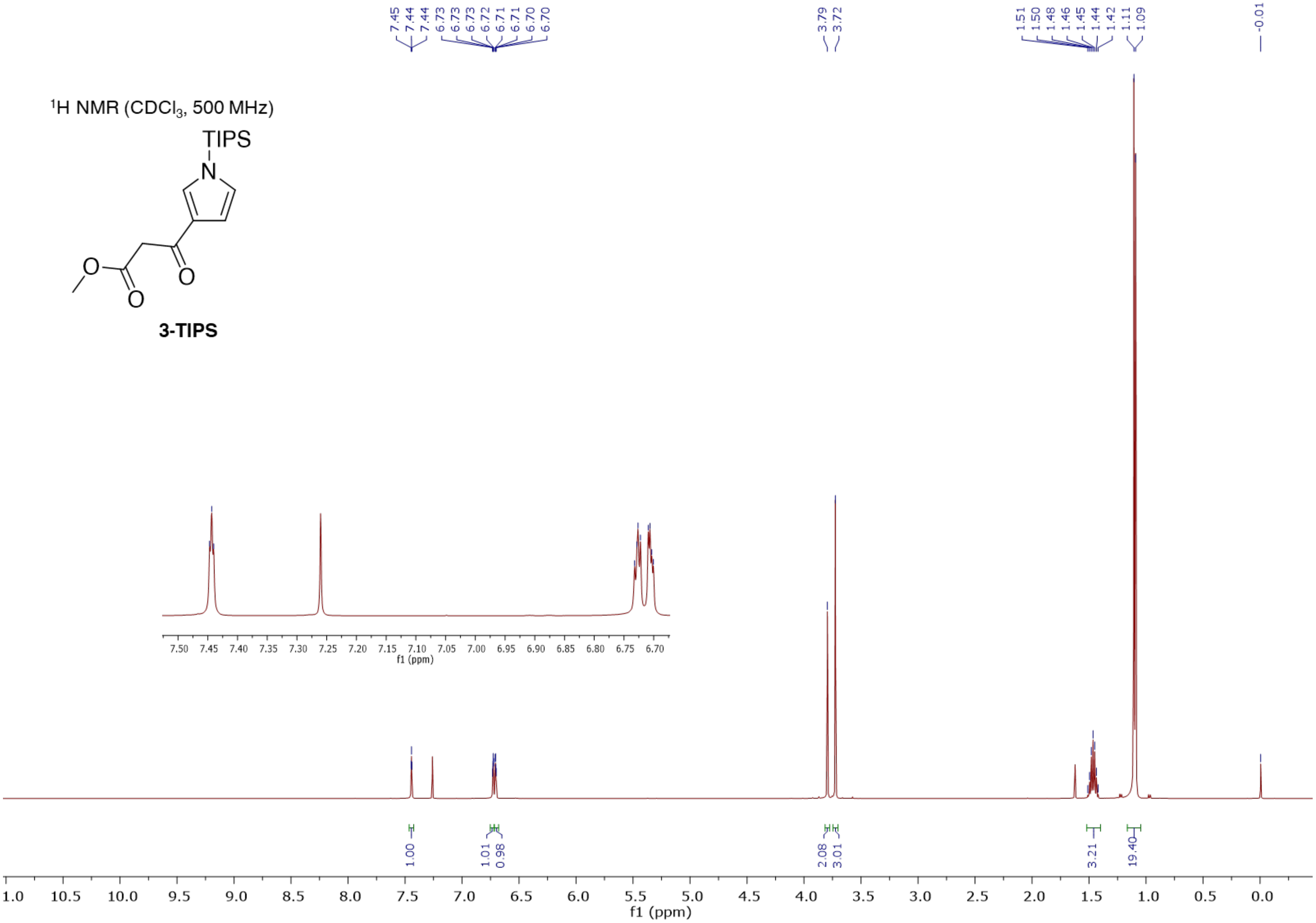

1H NMR (CDCl3, 500 MHz)
TIPS
N
O
O
O
3-TIPS
f1 (ppm)

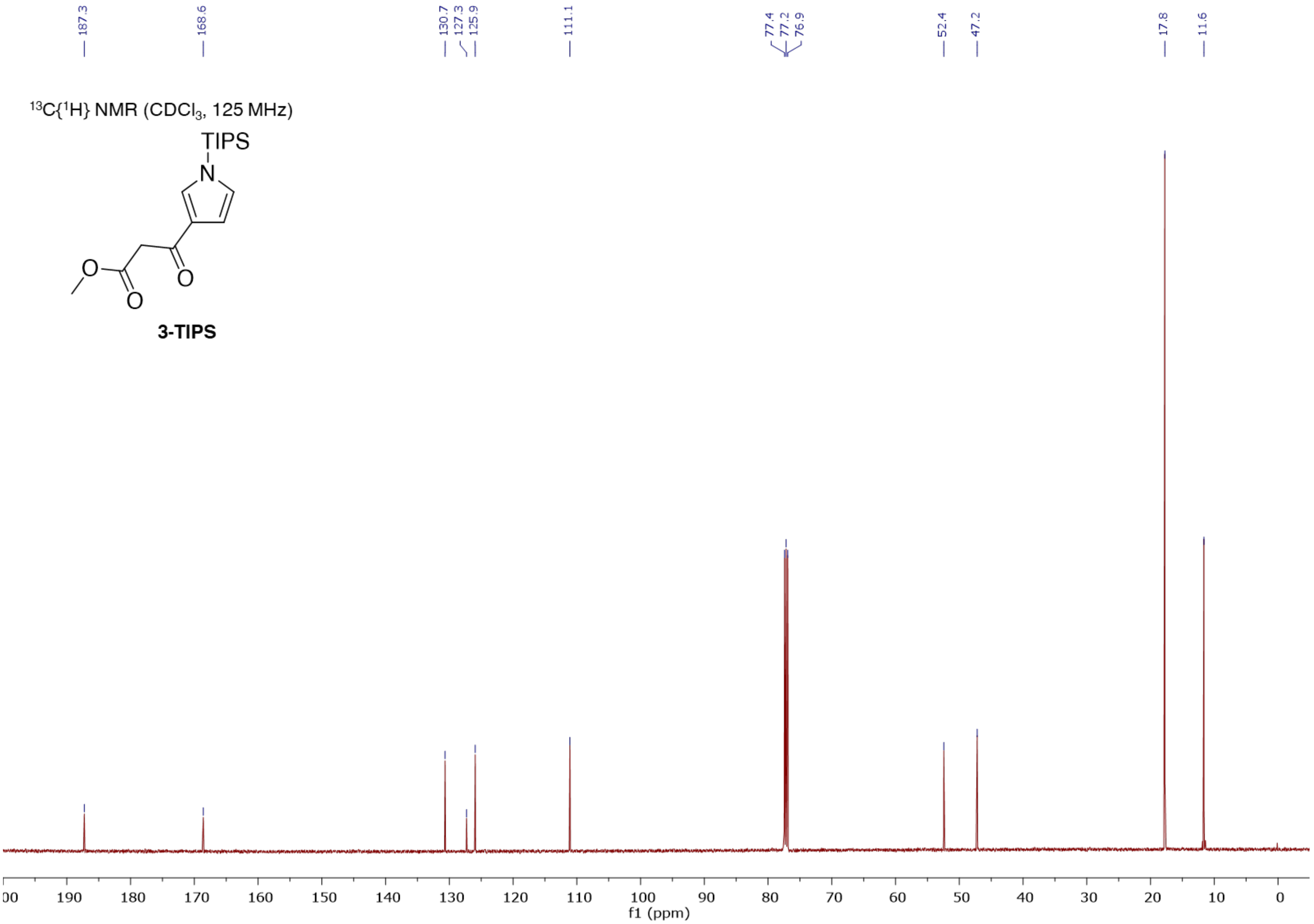

187.3
168.6
130.7
127.3
125.9
111.1
77.4
77.2
76.9
52.4
47.2
17.8
11.6
$^{13}C\{^1H\}$ NMR ($CDCl_3$, 125 MHz)
TIPS
N
O
O
O
3-TIPS
00
190
180
170
160
150
140
130
120
110
100
90
80
70
60
50
40
30
20
10
0
f1 (ppm)
